# New Insights Into the Backbone Phylogeny and Character Evolution of *Corydalis* (Papaveraceae) Based on Plastome Data

**DOI:** 10.3389/fpls.2022.926574

**Published:** 2022-08-05

**Authors:** Xiaodong Xu, Xuexiu Li, Dong Wang

**Affiliations:** ^1^School of Life Sciences, Central China Normal University, Wuhan, China; ^2^Bio-Resources key Laboratory of Shaanxi Province, Shaanxi University of Technology, Hanzhong, China

**Keywords:** *Corydalis*, backbone phylogeny, plastome rearrangement, character evolution, divergence time

## Abstract

A robust backbone phylogeny is fundamental for developing a stable classification and is instructive for further research. However, it was still not available for *Corydalis* DC., a species-rich (> 500 species), ecologically and medically important, but taxonomically notoriously difficult genus. Here, we constructed backbone phylogeny and estimated the divergence of *Corydalis* based on the plastome data from 39 *Corydalis* species (32 newly sequenced), which represent ca. 80% of sections and series across this genus. Our phylogenetic analyses recovered six fully supported main clades (I–VI) and provided full support for the majority of lineages within *Corydalis*. Section *Archaeocapnos* was unexpectedly turned out to be sister to the rest of the subg. *Corydalis* s. l. (clades IV–VI), thus treating as a distinct clade (clade III) to render all the main clades monophyletic. Additionally, some unusual plastome structural rearrangements were constantly detected within *Corydalis* and were proven to be lineage-specific in this study, which, in turn, provided further support to our phylogeny. A segment containing five genes (*trnV-UAC*–*rbcL*) in the plastome's LSC region was either normally located downstream of the *ndhC* gene in clade I species or translocated downstream of the *atpH* gene in clade II species or translocated to downstream of the *trnK-UUU* gene in clade III–VI species. The unique large inversion (ca. 50 kb) in the plastome LSC region of clade III species, representing an intermediate stage of the above translocation in clades IV–VI, firmly supported clade III as a distinct and early diverged clade within this large lineage (clades III–VI). Our phylogeny contradicted substantially with the morphology-based taxonomy, rejected the treatment of tuberous species as an independent evolutionary group, and proved that some commonly used diagnostic characters (e.g., root and rhizome) were results of convergent evolution, suggestive of unreliability in *Corydalis*. We dated the origin of crown *Corydalis* to the early Eocene (crown age 49.08 Ma) and revealed possible explosive radiation around 25 Ma, coinciding with the drastic uplift of the Qinghai-Tibetan Plateau in Oligocene and Miocene. This study provided the most reliable and robust backbone phylogeny of *Corydalis* to date and shed some new insights on the evolution of *Corydalis*.

## Introduction

*Corydalis* DC. is the largest genus within Papaveraceae, containing over 500 species (Catalogue of Life, 2022) divided into ca. 40 sections (Zhang et al., 2008). It is broadly distributed in northern temperate regions but are particularly diverse in China, especially in the Qinghai-Tibet Plateau (Lidén, 1986; Wu et al., 1999; Zhang et al., 2008). *Corydalis* species have displayed extensive morphological diversification and adaptation to diverse habitats (riversides, forests, shrubs, grasslands, screes, cliffs, etc.) from near sea level to more than 6,000 meters in elevation, which is of great interest to the evolutionary biologists and ecologists (Ohara and Higashi, 1994; Ohkawara et al., 1997; Kudo et al., 2001; Ehlers and Olesen, 2004; Zhang et al., 2013; Niu et al., 2014, 2017; Zhu et al., 2018). Additionally, a large number of species in *Corydalis* are medicinally valuable, and some have shown a great potential for anti-hepatitis, antitumor, treating cardiovascular diseases and releasing pains, such as the famous Chinese herb “Yuanhu” (*C. yanhusuo*; Luo et al., 1984; Editorial Board of Chinese Tibetan medicine, 1996; Kim et al., 1999; Chlebek et al., 2011; Chinese Pharmacopoeia Commission, 2015; Zhang B. et al., 2016; Alhassen et al., 2021; Deng et al., 2021). However, the classification of *Corydalis* is still controversial and notoriously difficult due, at least in part, to its intensive differentiation, complex morphological characters, and narrow distribution of enormously high elevation species. Despite the accumulation of knowledge toward clarifying the taxonomy and phylogeny of *Corydalis* during the past decades, a robust backbone phylogeny of *Corydalis* remains unresolved, which greatly hindered our in-depth exploitation of its evolution and utilization.

The genus *Corydalis* was formally established in 1805 by Candolle, with the inclusion of four species that were previously circumscribed in *Fumaria* (de Candolle, 1805). Since then, enormous new species were described from *Corydalis* (Persoon, 1806; de Candolle, 1821; Fedde, 1924, 1926; Su, 1980; Wu and Zhuang, 1982), and some infrageneric classifications were put forward (de Candolle, 1821; Fedde, 1936; Su and Wu, 1985). The first relatively comprehensive synopsis of *Corydalis* was provided by Lidén (1986), who classified the 250–300 *Corydalis* species, known in that age, into 19 sections. A decade later, Wu et al. (1996) proposed another detailed evolutional system and classified the ca. 400 known *Corydalis* species into two groups (*Corydalis* group and *Pistolochia* group) and 40 sections, which differed substantially from Lidén's (1986) treatment, particularly in the recognition and demarcation of sections. Almost at the same time, Lidén et al. (1995, 1997) applied ITS and *rps16* sequence of about 20 species to illustrate the phylogeny of *Corydalis* and divided this genus into three subgenera, i.e., subg. *Cremnocapnos*, subg. *Sophorocapnos*, and subg. *Corydalis*. Later, Wang (2006), based on the *rps16* and *matK* sequences of about 100 *Corydalis* species, further divided Lidén's subg. *Corydalis* (thereafter subg. *Corydalis* s. l.) into three subgenera, i.e., subg. *Corydalis* sensu stricto (thereafter subg. *Corydalis* s. str.), subg. *Rapiferae*, and subg. *Fasciculatae*; thus, in total, recognizing five subgenera within *Corydalis*. Although Wang's (2006) research is implicational for the infrageneric classification of *Corydalis*, it remains problematic because of the insufficient supporting information, low resolution, and the polyphyletic status of subg. *Corydalis* s. str. Probably referring to the results of previous phylogenetic analyses based on a few DNA markers, Zhang et al. (2008) circumscribed *Corydalis* species into three subgenera (subg. *Cremnocapnos*, subg. *Sophorocapnos*, and subg. *Corydalis* s. l.), 40 sections, and five series. Because China is the diversity center of *Corydalis*, although Zhang et al.'s (2008) classification mainly focuses on the Chinese species and neglected two sections (sect. *Filicinae* and sect. *Radixcava*) that are not recorded in China, it was still the most comprehensive *Corydalis* classification system to date. However, the systematic relationships of some *Corydalis* sections and series, particularly from subg. *Corydalis* s. l., in Zhang et al.'s (2008) classification system, remain unresolved. Some morphologically similar and systematically closely arranged sections were probably unnaturally related because of the convergent evolution of the root morphology that was emphasized before. In recent years, with the inclusion of several new DNA markers (*rbcL, psbA*-*trnH, trnG* intron, and *NADPH* gene), researchers have successfully resolved the phylogenetic position of some *Corydalis* species and investigated the utility of DNA markers within *Corydalis* (Zhang Z. X. et al., 2016; Jiang et al., 2018; Ren et al., 2018; Xu and Wang, 2018), while the relationship of *Corydalis* sections has not been focused on and remains controversial. Given that the previous classification systems of *Corydalis* are still controversial and unresolved so far, constructing a reliable and robust phylogeny to facilitate *Corydalis* classification remains one of the greatest necessities.

Plastome data have been proven to be powerful tools for resolving longstanding controversies at different taxonomic levels and for constructing reliable backbone phylogenies (Jansen et al., 2007; Moore et al., 2007, 2010; Ma et al., 2014; Barrett et al., 2016; Zhang et al., 2017; Li H. T. et al., 2019; Xu et al., 2019; Zhai et al., 2019; Zhao et al., 2021) because of their uniparental inheritance, moderate nucleotide substitution rates, abundant informative sites, and easy sequencing and assembling (Clegg et al., 1994; Jansen and Ruhlman, 2012). Meanwhile, rare plastome structural rearrangements have the potential to be useful phylogenetic markers because they are easily identified and typically lack homoplasy (Jansen and Palmer, 1987; Downie and Palmer, 1992; Doyle et al., 1996; Cosner et al., 2004). Surprisingly, limited plastomes to date are available for *Corydalis* species. Except for the seven plastomes from our previous studies (Xu and Wang, 2020, 2021; Yu et al., 2021; Huang et al., 2022), only one *Corydalis* plastome was formally and trustfully reported (Wu et al., 2020). This is not only in striking contrast to a species-rich genus with more than 500 species but also to the attraction they have for scientific interest.

In this study, we used plastome data from 39 *Corydalis* species (32 newly sequenced) representing ca. 80% of sections and series of this genus to construct a robust backbone phylogeny for *Corydalis*. Based on this, we further (1) inferred the phylogenetic relationships among the sections and series of *Corydalis*; (2) estimated the divergence times of *Corydalis* lineages; and (3) evaluated the phylogenetic implications of some characters, such as root and rhizome, which have been emphasized in the previous morphology-based classifications.

## Materials and Methods

### Taxon Sampling, DNA Extraction, Library Construction, and Sequencing

We newly sampled and sequenced the plastome of 32 *Corydalis* species in this study. Another seven *Corydalis* plastomes were also included, encompassing six from our previous studies (Xu and Wang, 2020, 2021; Huang et al., 2022), and one from the study of Wu et al. (2020). In total, the in-group sampling included 39 *Corydalis* species from 31 sections and four series (Table 1), representing about 80% of sections and series of *Corydalis* according to the infrageneric taxonomic system of Zhang et al. (2008). The other already reported *Corydalis* plastomes (Kanwal et al., 2019; Liu et al., 2021; Ren et al., 2021; Wang et al., 2021) were not included in this study because we have some concerns about the reliability of their plastome data. As we mainly focused on constructing the backbone phylogeny of *Corydalis*, our samplings were designed to cover as many sections as possible; thus, we mostly selected one representative for each section. Section *Strictae* and sect. *Archaeocapnos* each represent a main clade; thus, we sampled two species for each section to better represent the two clades. Section *Fumarioides* was further divided into two groups, so we sampled one species for each group. All the newly sequenced *Corydalis* samples in this study were collected from their wild populations and were eventually identified following the treatment of *Corydalis* in *Flora of China* (Zhang et al., 2008). The voucher specimens and DNA samples were deposited in the herbarium of Central China Normal University (CCNU), Wuhan, China. The procedures of DNA extraction, library preparation, and Illumina sequencing were the same as Xu and Wang (2021).

**Table 1:** Taxa, vouchers/references, and GenBank accession numbers used in phylogenetic analyses.

**Family**	**Species**	**Section/Series/Group**	**Voucher/References**	**GenBank accession**
Papaveraceae	***Corydalis melanochlora*** **Maxim**.	ser. *Clavatae*	Deqing, Yunnan, Wang et al., 160426	ON152781
	***C. pseudoadoxa*** **(C.Y. Wu. and H. Chuang) C.Y. Wu and H. Chuang**	ser. *Fusiformes*	Linzhi, Xizang, Wang et al., 180170	ON152797
	***C. minutiflora*** **C.Y. Wu**	ser. *Kokianae*	Yajiang, Sichuan, Wang et al., 160315	ON152779
	***C. curviflora*** **Maxim**.	ser. *Curviflorae*	Diebu, Gansu, Wang et al., 140382	ON152771
	***C. jingyuanensis*** **C.Y. Wu and H. Chuang**	sect. *Ellipticarpae*	Foping, Shaanxi, Wang et al., 170045	ON152787
	***C. elata*** **Bur. and Franch**.	sect. *Elatae*	Kangding, Sichuan, Wang et al., 170126	ON152788
	***C. hamata*** **Franch**.	sect. *Hamatae*	Dege, Sichuan, Wang et al., 180188	ON152799
	***C. petrophila*** **Franch**.	sect. *Priapos*	Deqing, Yunnan, Wang et al., 160427	ON152782
	***C. mucronata*** **Franch**.	sect. *Mucronatae*	Baoxing, Sichuan, Wang et al., 180126	ON152793
	***C. incisa*** **(Thunb.) Pers**.	sect. *Incisae*	Yifeng, Jiangxi, Wang et al., 180006	ON152790
	*C. temulifolia* Franch.	sect. *Asterostigma*	Huang et al., 2022	MT920558
	***C. brevirostrata*** **C.Y. Wu and Z.Y. Su**	sect. *Vermiculares*	Qumalai, Qinghai, Wang et al., 140435	ON152772
	***C. bungeana*** **Turcz**.	sect. *Chinenses*	Yanan, Shaanxi, Wang et al., 190014	ON152800
	***C. wuzhengyiana*** **Z.Y. Su and Lidén**	sect. *Chrysocapnos*	Basu, Xizang, Wang et al., 180174	ON152798
	*C. dasyptera* Maxim.	sect. *Chrysocapnos*	Wu et al., 2020	NC_047208
	***C. hendersonii*** **Hemsl**.	sect. *Latiflorae*	Songduo, Xizang, Wang et al., 140583	ON152774
	***C. casimiriana*** **subsp**. ***brachycarpa*** **Lidén**	sect. *Himalayanae*	Cuona, Xizang, Wang et al., 150281	ON152777
	***C. cornuta*** **Royle**	sect. *Ramososibiricae*	Jilong, Xizang, Wang et al., 150246	ON152776
	***C. borii*** **C.E.C. Fisch**.	sect. *Geraniifoliae*	Yadong, Xizang, Wang et al., 140642	ON152775
	***C. crispa*** **Prain**	sect. *Radicosae*	Songduo, Xizang, Wang et al., 140580	ON152773
	***C. trachycarpa*** **Maxim**.	sect. *Trachycarpae*	Xinlong, Sichuan, Wang et al., 160352	ON152780
	*C. inopinata* Prain ex Fedde.	sect. *Mucroniferae*	Xu and Wang, 2020	MT755641
	***C. fargesii*** **Franch**.	sect. *Fumarioides, ochotensis* group	NingShan, Shaanxi, Wang et al., 160443	ON152783
	***C. pseudoimpatiens*** **Fedde**	sect. *Fumarioides, sibirica* group	Zhuoni, Gansu, Wang et al., 140353	ON152770
	*C. davidii* Franch.	sect. *Davidianae*	Xu and Wang, 2021	MT920560
	***C. decumbens*** **(Thunb.) Pers**.	sect. *Duplotuber*	Wuhan, Hubei, Wang et al., 170001	ON152784
	***C. livida*** **Maxim**.	sect. *Flaccidae*	Guazhou, Gansu, Wang et al., 180133	ON152794
	***C. benecincta*** **W.W. Sm**.	sect. *Benecinctae*	Kangding, Sichuan, Wang et al., 160302	ON152778
	***C. caudata*** **(Lam.) Pers**.	sect. *Corydalis*	Haidian, Beijing, Wang et al., 170013	ON152786
	***C. retingensis*** **Ludlow**	sect. *Oocapnos*	Basu, Xizang, Wang et al., 180156	ON152796
	*C. hsiaowutaishanensis* T.P. Wang	sect. *Dactylotuber*	Xu and Wang, 2021	MT920561
	***C. anthriscifolia*** **Franch**.	sect. *Archaeocapnos*	Tianquan, Sichuan, Wang et al., 180123	ON152792
	***C. longicalcarata*** **H. Chuang and Z.Y. Su**	sect. *Archaeocapnos*	Hanyuan, Sichuan, Wang et al., 180096	ON152791
	***C. edulis*** **Maxim**.	sect. *Aulacostigma*	Nanzheng, Shaanxi, Wang et al., (1304)057	ON152801
	*C. saxicola* Bunting	sect. *Thalictrifoliae*	Xu and Wang, 2021	MT920562
	***C. balansae*** **Prain**	sect. *Sophorocapnos*	Changsha, Hunan, Wang et al., 180001	ON152789
	***C. racemosa*** **(Thunb.) Pers**.	sect. *Cheilanthifoliae*	Tongshan, Hubei, Wang et al., 170011	ON152785
	*C. adunca* Maxim.	sect. *Strictae*	Xu and Wang, 2021	MT920559
	***C. stricta*** **Stephan ex Fisch**.	sect. *Strictae*	Guazhou, Gansu, Wang et al., 180134	ON152795
	*Lamprocapnos spectabilis* (L.) Fukuhara.	–	Park et al., 2018	NC_039756
	*Chelidonium majus* L.	–	Shi et al., 2019	NC_046829
	*Coreanomecon hylomeconoides* Nakai	–	Kim and Kim, 2016	NC_031446
	*Hylomecon japonica* (Thunb.) Prantl. and Kündig	–	Zhang et al., 2019	NC_045388
	*Macleaya microcarpa* (Maxim.) Fedde	–	Zeng et al., 2018	NC_039623
	*Meconopsis racemosa* Maxim.	–	Zeng et al., 2018	NC_039625
	*Papaver somniferum* L.	–	Sun et al., 2016	NC_029434
Circaeasteraceae	*Kingdonia uniflora* Balf. f. et W.W. Smith	–	Sun et al., 2017	NC_035873
Lardizabalaceae	*Akebia quinata* (Thunb. ex Houtt.) Decne.	–	Li et al., 2016	NC_033913
Menispermaceae	*Stephania japonica* (Thunb.) Miers.	–	Sun et al., 2016	NC_029432
Ranunculaceae	*Glaucidium palmatum* Siebold and Zucc.	–	Zhai et al., 2019	NC_041539
	*Ranunculus cantoniensis* DC.	–	Li T. J. et al., 2019	NC_045920
Berberidaceae	*Mahonia bealei* (Fort.) Carr.	–	Ma et al., 2013	NC_022457
	*Nandina domestica* Thunb.	–	Moore et al., 2006	NC_008336
Eupteleaceae	*Euptelea pleiosperma* Hook. f. et Thoms.	–	Sun et al., 2016	NC_029429
Buxaceae	*Buxus microphylla* Siebold and Zucc.	–	Hansen et al., 2007	NC_009599
Vitaceae	*Vitis rotundifolia* Michx.	–	Lynch and Kane, 2014	NC_023790
Trochodendraceae	*Trochodendron aralioides* Siebold and Zucc.	–	Sun et al., 2013	NC_021426
Sabiaceae	*Sabia yunnanensis* Franch.	–	Sun et al., 2016	NC_029431
Ceratophyllaceae	*Ceratophyllum demersum* L.	–	Moore et al., 2007	NC_009962
Acoraceae	*Acorus gramineus* Soland.	–	Zhu et al., 2016	NC_026299

*The species in bold were newly sequenced*.

In addition to the above *Corydalis* samples, we included 21 species (Table 1) as outgroups, including 16 representative species from Ranunculales, three species from the rest of the three early-diverging eudicots lineages (*Sabia yunnanensis* Franch., NC_029431, Sabiaceae; *Trochodendron aralioides* Siebold & Zucc., NC_021426, Trochodendraceae; and *Buxus microphylla* Siebold & Zucc., NC_009599, Buxaceae), one core eudicots species (*Vitis rotundifolia* Michx., NC_023790, Vitaceae), one species from Ceratophyllales (*Ceratophyllum demersum* L., NC_009962, Ceratophyllaceae), and one monocots (*Acorus gramineus* Soland., NC_026299, Acoraceae). Plastome sequences of outgroup species were downloaded from National Center for Biotechnology Information (NCBI). Details of the selected species, including their classification, voucher information, references, and Genbank accession numbers, are provided in Table 1.

### Plastome Assembly and Annotation

The procedure for assembly and annotation of the newly sequenced plastomes was followed according to the study of Xu and Wang (2021), with one alternation in the process of annotation using PGA (Qu et al., 2019), where we added the plastome of *C. inopinata* (MT755641) as the reference. All the newly annotated plastomes were submitted to NCBI, and the accession numbers are shown in Table 1.

The major plastome rearrangements within *Corydalis* species were detected using Mauve 2.4.0 (Darling et al., 2004) with the “progressiveMauve” algorithm. To improve the resolution of the figure, and because the species in the same clade share similar major plastome arrangements, only two representative species from each *Corydalis* clade were included, and the successfully assembled plastomes were preferentially chosen. Finally, 12 *Corydalis* species, that are *C. stricta, C. adunca, C. racemosa axicola, C. saxicola, C. longicalcarata, C. anthriscifolia, C. hsiaowutaishanensis, C. livida, C. davidii, C. dasyptera, C. temulifolia*, and *C. pseudoadoxa*, and the outgroup species *Papaver somniferum* (NC_029434), which showed conserved plastome structure, were used in Mauve analyses. The schematic diagrams of the plastome LSC region of *C. adunca, C. longicalcarata*, and *C. hsiaowutaishanensis* were drawn in OGDRAW v1.3.1 (Greiner et al., 2019) and adjusted manually to display the possible process of the translocation event in *Corydalis* plastomes.

### Phylogenetic Analyses

In total, 60 plastomes were included in our phylogenetic analyses, including all the 39 *Corydalis* plastomes and 21 outgroup plastomes (Table 1). Among them, *Acorus gramineus* (NC_026299), which was the sister to the rest of the species, was used as an outgroup to root the trees. The data matrix contains coding sequences (CDS) of 64 protein-coding genes, which were shared among the 60 plastomes. The other plastome protein-coding genes were excluded because they have been lost or pseudogenized in one or more analyzed plastomes. Two methods, i.e., Bayesian Inference (BI) and Maximum Likelihood (ML), were used to infer the phylogenetic relationships within *Corydalis*. The procedures from protein-coding DNA sequence extraction to phylogenetic tree construction were followed according to the study of Xu and Wang (2021), with a few alternations. In this study, the ambiguous regions in the aligned sequences were trimmed using trimAl v.14 (Capella-Gutiérrez et al., 2009) to remove all columns with gaps in more than 20% of the sequences (-gt 0.8). The Markov chains were increased to 82 million generations in the BI analysis using MrBayes v3.2.7 (Ronquist et al., 2012), and the convergences between the runs were inspected with Tracer v.1.7 (Rambaut et al., 2018) to ensure that the effective sampling sizes (ESS) for all relevant estimated parameters were above 200. For the ML analysis using RAxML v8.2.12 (Stamatakis, 2014), we used the GTRGAMMAI model. Trees produced in this study were visualized using the program FigTree v.1.4.4 (Rambaut, 2018).

### Molecular Dating Analyses

We used the BEAST v2.6.3 package (Bouckaert et al., 2019) to estimate the divergence times of *Corydalis* lineages. The lognormal relaxed clock (uncorrelated) model was used to account for rate variability among lineages. The speciation model was set as the Yule speciation model. The optimal nucleotide substitution model (GTR substitution model and four rate categories) was determined by jModeltest v2.1.10 (Darriba et al., 2012) using the corrected Akaike Information Criterion (AICc). Hindered by the lack of accurate, undisputed, and informative fossils for *Corydalis*, we followed a two-step calibration strategy to constrain the divergence of *Corydalis*. The data matrixes were newly prepared for each BEAST analysis; CDS sequences of 64 shared protein-coding genes were extracted using Biopython v1.77 (Cock et al., 2009), aligned directly using MAFFT v7.450 (Katoh and Standley, 2013), trimmed using trimAl v.14 (-gt 0.8; Capella-Gutiérrez et al., 2009), and finally concatenated using Biopython v1.77.

To utilize the fossil from outgroups, an initial analysis, including all the 21 outgroup species (Table 1) and one representative from each of the six *Corydalis* clades (*C. stricta, C. racemosa, C. anthriscifolia, C. hsiaowutaishanensis, C. davidii*, and *C. bungeana*), was conducted to obtain the crown age of *Corydalis*. The tricolpate pollen fossils (Hughes and McDougall, 1990; Doyle and Hotton, 1991; Hughes, 1994) and flower fossil of *Teixeiraea lusitanica* von Balthazar with affinities to the Ranunculales (von Balthazar et al., 2005), which were thought to be reliable and commonly used in previous research (Magallón et al., 2015; Li H. T. et al., 2019; Yang et al., 2020), were used as age constraints in this initial analysis. The crown age of eudicot (Node1) was constrained to a minimum of 125 million years ago (hereafter abbreviated as Ma; log-normal distribution, mean = 1, SD = 1.25, offset = 125, and 95% HPD: 125–146) based on the tricolpate pollen fossils. The crown age of Ranunculales (Node2) was constrained to a minimum of 112 Ma (log normal distribution, mean = 1, SD = 1.25, offset = 112, and 95% HPD: 112–133) based on the flower fossil of *Teixeiraea lusitanica*. The start tree was set as the random tree in this initial analysis.

In the second BEAST analysis, we included all the *Corydalis* species and only three outgroup species (*Lamprocapnos spectabilis*, NC_039756; *Papaver somniferum*, NC_029434; and *Akebia quinata*, NC_033913) to obtain the detailed divergence time for *Corydalis* lineages. For this phylogeny calibration, we used the dates from the first BEAST analysis as calibration points. The crown ages for subfam. Fumarioideae and *Corydalis* were set to 65.62 Ma (normal distribution, mean = 65.62, Sigma = 1, offset = 0, and 95% HPD: 64–67.3) and 49.23 Ma (normal distribution, mean = 9.23, Sigma = 1, offset = 0, and 95% HPD: 47.6–50.9), respectively. The ML tree constructed using RAxML v8.2.12 (Stamatakis, 2014) based on the second BEAST analysis data matrix was used as the starting tree.

The Markov chain Monte Carlo (MCMC) simulation was run for 8.8 × 10^8^ generations and 9.5 × 10^8^ generations for the initial and second BEAST analyses respectively, with sampling for every 1,000 generations. Convergences between the runs were inspected by Tracer v.1.7 (Rambaut et al., 2018) to ensure all the ESS were above 200. TreeAnnotator v1.8.4 (implemented in BEAST tools package) was used to summarize the tree results with a burn-in of 10%. The stratigraphic boundaries were in compliance with the International Chronostratigraphic Chart (International Commission on Stratigraphy, https://stratigraphy.org/, v2020/03).

## Results

### Sequencing and Assembling Results

Our sequencing generated 173.69 G raw base (Q30 > 88.84%) from the 32 newly sequenced *Corydalis* species. For each species, 2.96–8.33 G raw base (12,766,960–27,770,716 raw 150 nt paired-end reads) were obtained (Supplementary Table 1). In the GetOrganelle assemblies, only five plastomes were successfully and completely assembled into a circular genome. For the other 27 plastomes, due to the existence of some repetitive sequences, the GetOrganelle assembled graph was intertwined in some nodes and could not be disengaged without uncertainty; thus, we just extracted the unambiguous part into 5–10 scaffolds (Supplementary Table 1) to conduct the rest of the analyses. Although the plastomes of these species were not successfully assembled into a circular genome, most of the gene regions were successfully assembled in the extracted scaffolds, except for 15 genes that involve rearrangement (*accD, trnN-GUU, trnV-UAC*, and 11 *ndh* genes) or near the intertwined node (*trnK-UUU*) that were incomplete or lost for some species. After adjustment of order and direction according to the GetOrganelle assembly results, and by comparing with the reference (*C. inopinata*, MT755641), the plastome scaffolds were connected with 3 consecutive undetermined (“N”) bases for each species. The length of the five complete plastomes ranged from 173,581 bp (*C. pseudoimpatiens*) to 213,295 bp (*C. incisa*), whereas the assembled length (scaffolds in IR was counted two times and not including the “N”) of the 27 incomplete plastomes varied from ~164 kb (*C. casimiriana* subsp. *brachycarpa*) to ~205 kb (*C. retingensis*).

### Phylogenetic Relationships

The concatenated alignment of 64 plastome protein-coding genes yielded a data matrix of 53,769 nucleotide sites (13,133 nt, 24.4% were parsimony-informative) for 60 species (39 *Corydalis* and 21 outgroups). The optimal partitioning scheme that was determined using the MrBayes models was composed of 75 subsets and 12 substitution models (Supplementary Table 2) and was used in BI analyses. All the parameters in the BI analysis achieved an ESS of >200. The AICc values of another three independent PartitionFinder2 analyses using GTR, GTR + G, or GTR + I + G were 841,515.036798, 827,722.290587, and 822,822.338477, respectively. The partitioning scheme determined using GTR + I + G (65 subsets, Supplementary Table 3) had the lowest AICc value and was used in the ML analysis.

**Figure 1:**
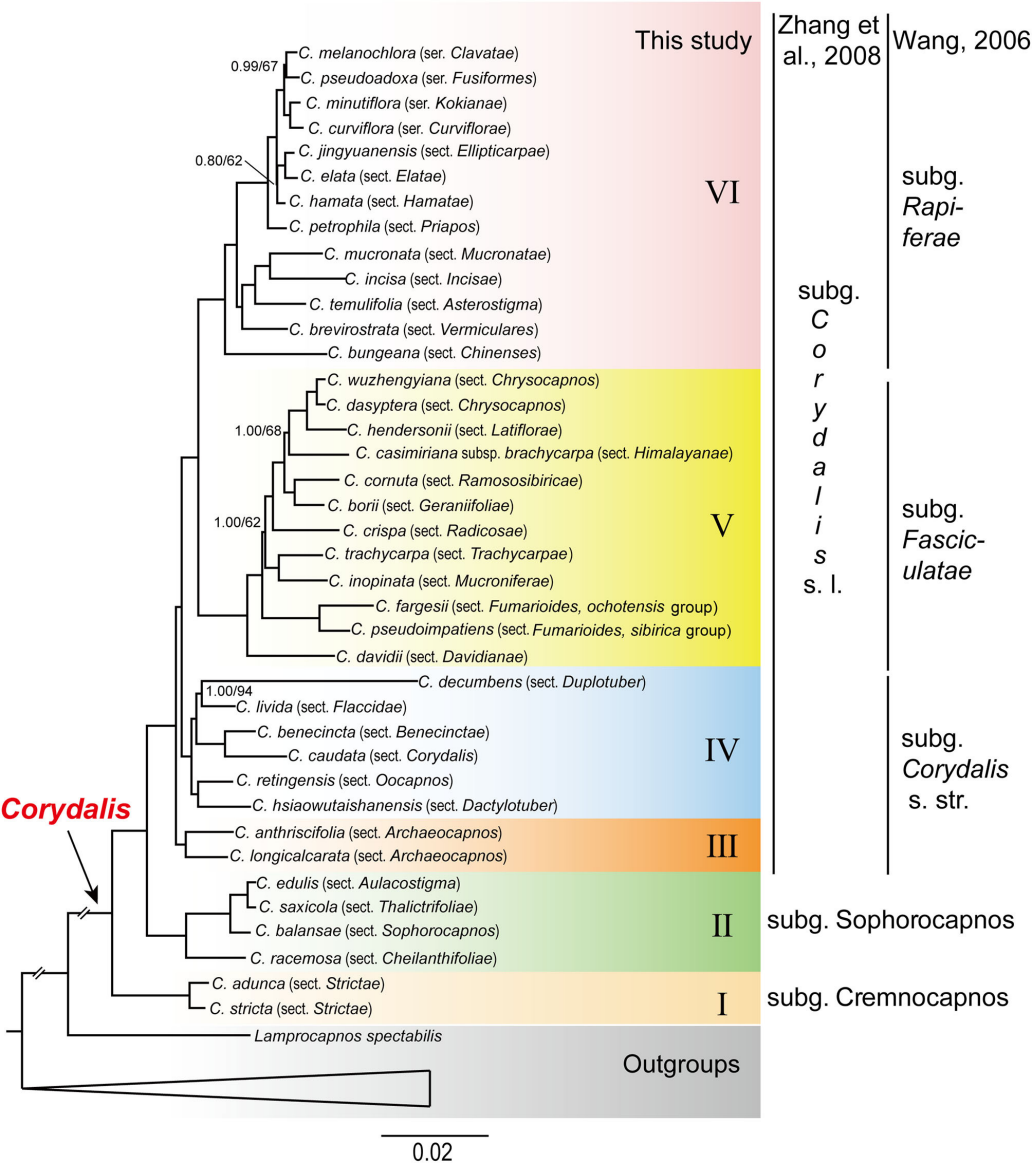
Phylogenetic tree conducted using BI methods. The numbers above branches represent BI posterior probability and ML bootstrap support, respectively. Clades have PP-value of 1 and BS-value of 100, unless otherwise indicated. The circumscription of subgenera just differed for clade III + IV + V + VI, the left was by Zhang et al. (2008), while the right was by Wang (2006).

In our study, the BI and ML analyses yielded identical phylogenetic tree topology (Figure 1), although the support values differed at several nodes. All the *Corydalis* species were grouped in a well-resolved monophyletic clade (PP = 1, BS = 100). Within *Corydalis*, six fully supported clades (clade I to VI; PP = 1, BS = 100) were recovered (Figure 1). Clade I was composed of only one section, i.e., sect. *Strictae* (*C. adunca* and *C. stricta*), and was strongly supported as the sister to the rest of *Corydalis*. Clade II was composed of four successively diverged sections, i.e., sect. *Cheilanthifoliae* (*C. racemosa*), sect. *Sophorocapnos* (*C. balansae*), sect. *Aulacostigma* (*C. edulis*), and sect. *Thalictrifoliae* (*C. saxicola*), and each sub-clade received full support (PP = 1, BS = 100). Clade III was composed of sect. *Archaeocapnos* (*C. anthriscifolia* and *C. longicalcarata*) alone, which was the sister to the rest of subg. *Corydalis* sensu lato. Clade IV was composed of six sections, i.e., sect. *Oocapnos* (*C. retingensis*), sect. *Dactylotuber* (*C. hsiaowutaishanensis*), sect. *Duplotuber* (*C. decumbens*), sect. *Flaccidae* (*C. livida*), sect. *Benecinctae* (*C. benecincta*), and sect. *Corydalis* (*C. caudate*), and from the first one, every two sections were grouped to be a monophyletic sub-clade. Except for the clade that was composed of sect. *Duplotuber* (*C. decumbens*) and sect. *Flaccidae* (*C. livida*), which received the support of 94% in the ML analyses (PP = 1, BS = 94), all the other sub-clades in clade IV received full support (PP = 1, BS = 100). Clade V was composed of 11 sections, i.e., sect. *Davidianae* (*C. davidii*), sect. *Fumarioides* (*C. fargesii* and *C. pseudoimpatiens*), sect. *Trachycarpae* (*C. trachycarpa*), sect. *Mucroniferae* (*C. inopinata*), sect. *Radicosae* (*C. crispa*), sect. *Latiflorae* (*C. hendersonii*), sect. *Chrysocapnos* (*C. wuzhengyiana* and *C. dasyptera*), sect. *Himalayanae* (*C. casimiriana* subsp. *brachycarpa*), sect. *Ramososibiricae* (*C. cornuta*), and sect. *Geraniifoliae* (*C. borii*). Except for the two nodes that received relatively low support (PP = 1, BS = 62; and PP = 1, BS = 68), all the rest of the sub-clades in clade V received full support (PP = 1, BS = 100). Clade VI was composed of nine sections and four series, i.e., sect. *Chinenses* (*C. bungeana*), sect. *Vermiculares* (*C. brevirostrata*), sect. *Asterostigma* (*C. temulifolia*), sect. *Mucronatae* (*C. mucronata*), sect. *Incisae* (*C. incisa*), sect. *Priapos* (*C. petrophila*), sect. *Ellipticarpae* (*C. jingyuanensis*), sect. *Elatae* (*C. elata*), sect. *Hamatae* (*C. hamata*), ser. *Clavatae* (*C. melanochlora*), ser. *Fusiformes* (*C. pseudoadoxa*), ser. *Kokianae* (*C. minutiflora*), and ser. *Curviflorae* (*C. curviflora*). Except for the two nodes that received relatively low support (PP = 0.80, BS = 62; and PP = 0.99, BS = 67), all the rest of the nodes in clade VI received full support (PP = 1, BS = 100).

### Plastome Rearrangements

**Figure 2:**
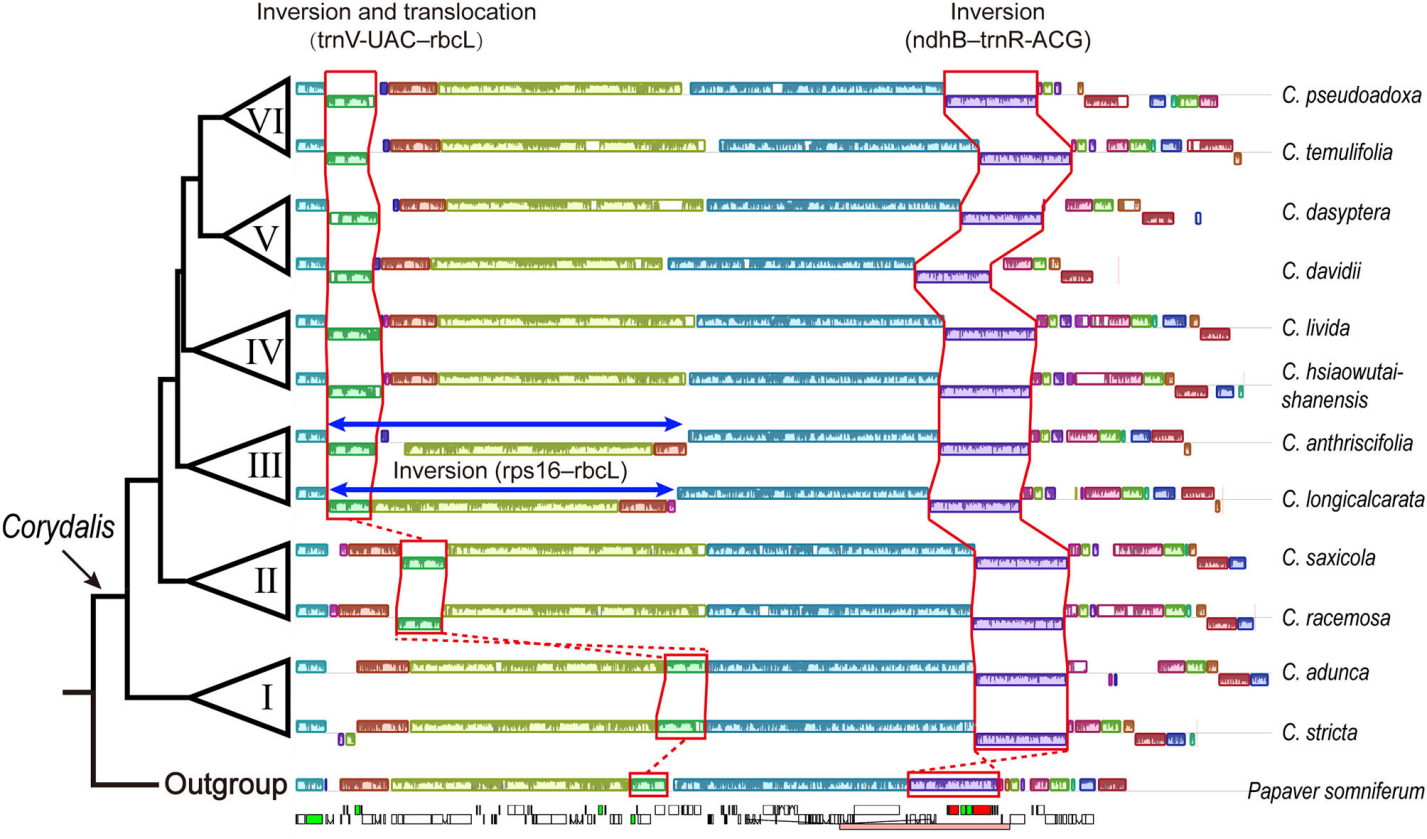
Major structural rearrangements in *Corydalis* plastome. For each species, only one IR region was included. Colored blocks represent locally collinear blocks (LCBs). Blocks drawn below the horizontal line indicate sequences found in an inverted orientation. The relevant genes and the range of IR region (pink boxes) were indicated in the outgroup plastome.

In the 32 newly sequenced and seven previously reported *Corydalis* plastomes, the segment, including *trnV-UAC*–*rbcL*, located downstream of the *ndhC* gene in all the two species from clade I, was translocated downstream of the *atpH* gene in all four species from clade II or was translocated to downstream of the *trnK-UUU* gene in 27 species of clade III + IV + V + VI (Figure 2). For the rest of the six *Corydalis* species (*C. fargesii, C. crispa, C. borii, C. cornuta, C. hendersonii*, and *C. wuzhengyiana*) that all come from clade V, although their scaffold which contains the *trnV-UAC–rbcL* genes were not connected successfully with the scaffold which contains *trnK-UUU* gene, we can also deduce with confidence that their *trnV-UAC*–*rbcL* genes have also translocated downstream of the *trnK-UUU* gene. Because their *trnV-UAC*–*rbcL* genes were upstream of *rps16* gene; and the plastome segments normally upstream and downstream of *trnV-UAC*–*rbcL*, i.e., *rps4*–*ndhC* and *psaI*–*ycf4*, were connected directly. For both species in clade III, we detected a ca. 50-kb large inversion in the LSC region, which includes 45 genes and spans from *rps16* to *rbcL*. For *C. anthriscifolia* (clade III), the segment including five genes (*rps16*–*trnS-GCU*) was inserted into the above large inversion, located downstream of the *trnV-UAC* gene.

**Figure 3:**
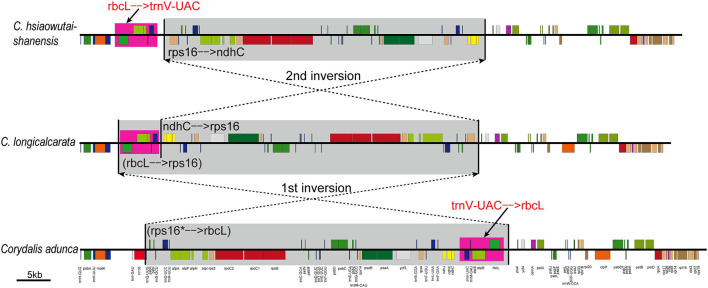
The two inversions detected in *Corydalis* plastomes LSC region that could explain the translocation of the segment involving *trnV-UAC*–*rbcL* in clade IV + V + VI. The inverted regions were highlighted with gray background, while the translocated regions were highlighted with pink background.

Based on the above evidence and from our *Corydalis* phylogeny, we deduced the translocation event in the LSC region of *Corydalis* plastome as the result of two overlap inversions. The translocation process shared by clade IV + V + VI species is taken as an example and illustrated in Figure 3. The first inversion inverted the *rps16*–*rbcL* region to its reversed direction, which was exactly detected in *C. longicalcarata* (in clade III), while the second inversion recovered merely the posterior part (*rps16*–*ndhC*) of the inverted region to its original direction, and the region that contains *trnV-UAC*–*rbcL* is still left downstream of the *trnK-UUU* gene.

An inversion involving the *ndhB*–*trnR-ACG* genes in the IR regions occurred consistently in all the *Corydalis* plastomes (Figure 2). This inversion is incontestable and can be directly observed in 37 *Corydalis* plastomes. For the rest of the two *Corydalis* plastomes, although the scaffold contains *ndhB*–*trnR-ACG* is not successfully connected with its neighboring scaffolds, we can also deduce that the inversion of *ndhB*–*trnR-ACG* has also occurred because of the direct connection of *ycf2*–*trnL-CAA*–*trnR-ACG*–*rrn4.5* in *C. borii*, the direct connection of *trnL-CAA*–*trnR-ACG*–*rrn4.5*, and the direct connection of *ndhB*–*trnN-GUU*–*ndhF* in *C. curviflora*.

### Divergence Time Estimation

**Figure 4:**
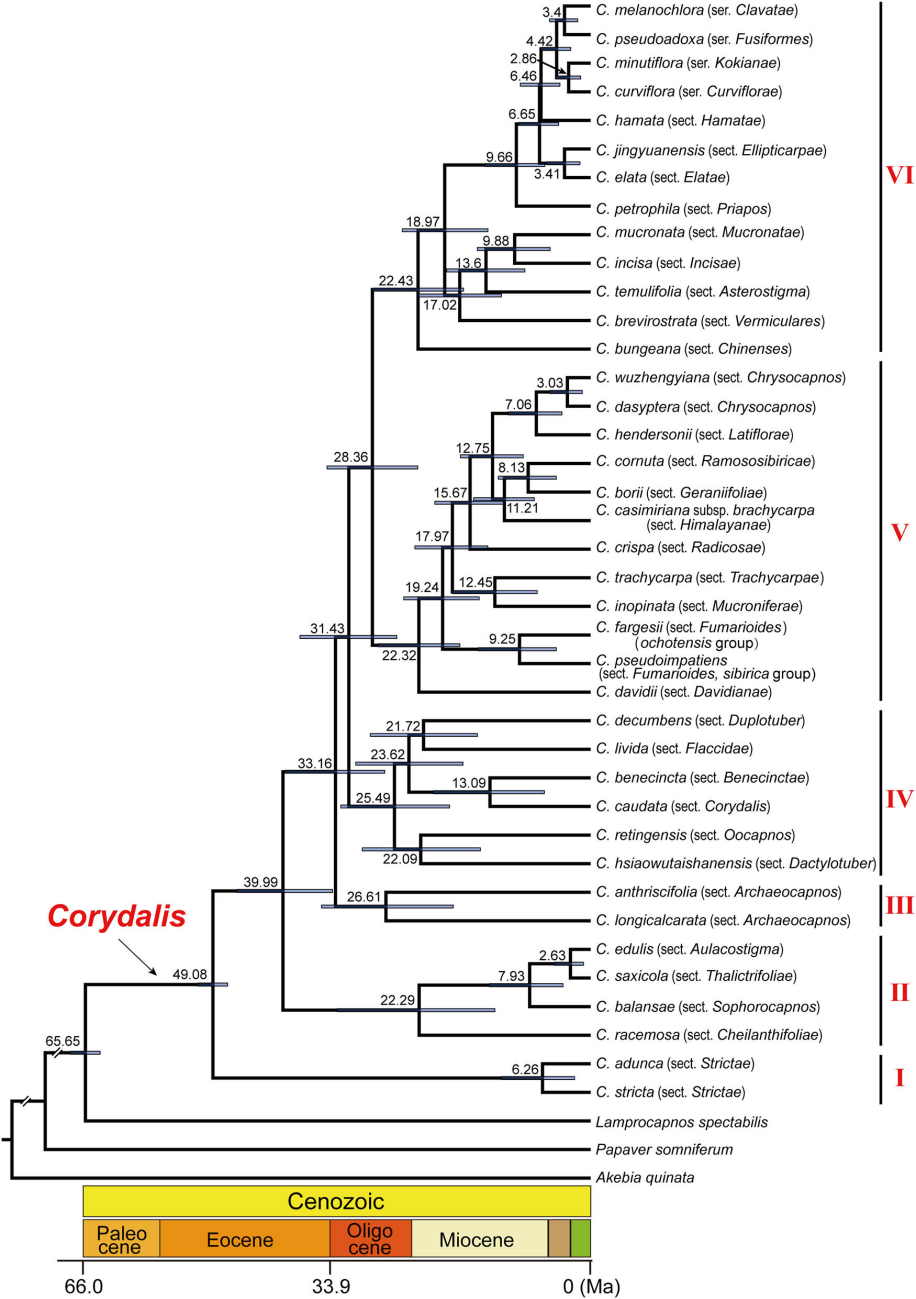
Detailed chronogram showing divergence time of *Corydalis* estimated in BEAST based on all the 39 *Corydalis* plastomes. Estimated mean ages are shown near the nodes, and blue bars represent 95% high posterior density.

The initial BEAST analysis (8.8 × 10^8^ generations MCMC, ESS ≥ 202) resulted in the divergence of *Corydalis* from the *Lamprocapnos* around 65.62 Ma (95% HPD: 85.15–0.34), and the crown age of *Corydalis* is around 49.23 Ma (95% HPD: 68.14–0.33; Supplementary Figure 1). In the second BEAST analysis (9.5 × 10^8^ generations MCMC, ESS ≥ 233; Figure 4), the split of *Corydalis* ancestors from *Lamprocapnos* was dated to 65.65 Ma (95% HPD: 67.60–63.72), and *Corydalis* began to diversify in early Eocene (crown age 49.08 Ma, 95% HPD: 51.03–47.18). The three *Corydalis* subgenera (corresponding to clades I, II, and III + IV + V + VI) emerged until late Eocene (39.99 Ma, 95% HPD: 45.96–33.54), while the six main lineages emerged until late Oligocene (28.36 Ma, 95% HPD: 34.25–22.46). During 26.61–22.29 Ma, all the species-rich clades within *Corydalis* radiated simultaneously and gradually formed the species that were distributed in different sections. The detailed divergence time of *Corydalis* lineages is presented in Supplementary Table 4.

## Discussion

### Phylogeny of *Corydalis*

In striking contrast to a genus with more than 500 species, relatively little attention has been paid to the phylogeny of *Corydalis*. Previous molecular phylogenetic analyses of *Corydalis*, despite having contributing to our understanding of the circumscription, classification, and phylogeny of this genus (Lidén et al., 1995, 1997; Pérez-Gutiérrez et al., 2015; Sauquet et al., 2015; Zhang Z. X. et al., 2016), were unable to construct a reliable backbone phylogeny for *Corydalis* due to the use of only a few DNA markers, inadequate sampling of representative lineages, or low resolution. Our study, based on plastome data of 39 species from ca. 80% of sections and series of *Corydalis*, shed new and robust insight into the backbone phylogeny of *Corydalis*.

Our plastome phylogeny of *Corydalis* recovered six fully supported monophyletic main clades (I–VI; PP = 1, BS = 100; Figure 1) within this genus, rather than five as previously suggested (Wang, 2006). Our results were much superior to Wang's (2006) cursory phylogeny using *rps16* and *matK* sequences by gaining full support for most of the clades within *Corydalis*. Four of the current clades (I, II, V, and VI) were consistent with Wang's (2006) four clades, while the third polyphyletic clade in Wang's (2006) phylogeny was composed of two monophyletic clades (III and IV). Clade III (sect. *Archaeocapnos*) was newly discovered as sister to the rest of the subg. *Corydalis* s. l. in our analyses and, thus, separated to render all the *Corydalis* main clades monophyletic. Clade IV was inarguably a better resolved monophyletic clade than that indicated in Wang's (2006) phylogeny, and all the relationships within clade IV were unambiguous (PP = 1, BS > 94). The morphologically similar and closely arranged sections [such as sect. *Trachycarpae* (clade V) and ser. *Curviflorae* (clade VI)] were highly supported to be separated into different main clades, and their relationship with other sections was well-resolved. As a whole, our phylogenetic analyses clearly revealed the relationship of most of the *Corydalis* sections (all the involved; ca. 80% of this genus) and successfully constructed a robust backbone phylogeny for *Corydalis*.

### Plastome Structural Rearrangements Provide Further Support for Our *Corydalis* Phylogeny

Our phylogeny is further confirmed by the unusual structural rearrangements detected in the *Corydalis* plastomes. Plastome structural rearrangements, which were thought to be rare within angiosperms (particularly for photosynthetic members, Palmer, 1985; Wicke et al., 2011; Ruhlman and Jansen, 2014; Mower and Vickrey, 2018), were found to be common within *Corydalis* species and showed significant systematic implications in this study.

The location of five genes (*trnV-UAC*–*rbcL*) in the plastome LSC region is lineage-specific in our phylogeny, which either resided normally downstream of the *ndhC* gene in all the clade I species or translocated downstream of the *atpH* gene in clade II species or translocated downstream of the *trnK-UUU* gene in clade III + IV + V + VI species (Figure 2). The conservative location of these five genes (*trnV-UAC*–*rbcL*) in clade I supported its early divergence within *Corydalis*. The translocation of these five genes (*trnV-UAC*–*rbcL*) was deduced to be the result of two overlap inversions in our previous study (Xu and Wang, 2021) and an intermediate plastome structure was supposed to have occurred in the evolutionary history of *Corydalis*. In this study, the intermediate inversion has been detected in clade III species; *C. longicalcarata* showed exactly the intermediate inversion of *rps16*–*rbcL* (ca. 50 kb; Figure 3), while *C. anthriscifolia* has probably undergone further inversions that translocated *rps16*–*trnS-GCU* to downstream of *rbcL*–*trnV-UAC*. This unique intermediate inversion in the plastome LSC region of clade III species distinguished them from the rest of the *Corydalis* species within this large lineage (clade III + IV + V + VI) and supported its primitive status within this lineage. The translocation in clade II species, although involved the same five genes (*trnV-UAC*–*rbcL*), was probably independently originated through another two overlap inversions: the first involved *atpI*–*rbcL* and the second involved *atpI*–*ndhC*. Conclusively, the unique plastome structure and rearrangements of *Corydalis* species have offered further substantial support for the monophyly of clades I, II, III, and IV + V + VI. As to clades IV, V, and VI, a more detailed research is needed to examine if some synapomorphies exist in their plastome that is in support of their respective monophyly.

The location of these five genes (*trnV-UAC*–*rbcL*) was in support of the division of *Corydalis* into three subgenera by Lidén et al. (1995, 1997) and Zhang et al. (2008), i.e., subg. *Cremnocapnos* (clade I), subg. *Sophorocapnos* (clade II), and subg. *Corydalis* s. l. (clade III + IV + V + VI). However, clade III is distinct within subg. *Corydalis* s. l., and the monophyly of clades IV, V, and VI are fully supported in our phylogenetic analyses; thus, a further division of this largest subgenera within *Corydalis* is needed. However, we would prefer awaiting and accumulating more extensive evidence to make this subdivision.

Another large inversion (ca. 13 kb, including 11 genes, from *ndhB* to *trnR-ACG*) in the IR region, although inverted uniformly throughout *Corydalis*, was probably not diagnostic for *Corydalis*. A similar inversion (from *rps7* to *trnR-ACG*) has been reported in the plastome IR region of *Lamprocapnos spectabilis* (Park et al., 2018), which differed from the *Corydalis* IR inversion just by the location of the *ndhB* gene. The *ndhB* gene is normally located downstream of the *rps7* gene in *Corydalis*, while translocated downstream of the *rps16* gene in the IR region of *L. spectabilis*. Considering the phylogenetic relationship of *Corydalis* and *Lamprocapnos* (both belonging to Fumarioideae) and their possible plastome evolution history, the seemingly unique IR inversion of *Corydalis* was possibly also shared by the common ancestor of these two genera. An extended sampling is needed to illustrate the evolutionary history of the IR inversion within Fumarioideae. These plastome rearrangements, although were found to be lineage-specific within this study, their utility as phylogenetic markers remain to be tested in other unsampled *Corydalis* species.

### Previous Classifications of *Corydalis* Were Unnaturally Arranged

There were three relatively complete classification systems of *Corydalis* before our study, by Lidén (1986), Wu et al. (1996, 1999), and Zhang et al. (2008), respectively, with the former two emphasizing solely on morphological characters, and the latter one referring to molecular phylogeny. Due to the complexity of evolution patterns (divergent, convergent, and reversal), the morphology-based classification system occasionally failed to elucidate the true phylogenetic relationships and is often unstable and unreliable. Consequently, these three relatively complete classification systems of *Corydalis* (Lidén, 1986; Wu et al., 1996, 1999; Zhang et al., 2008) not only differed considerably from each other but also contradicted a lot with our plastome data-based phylogeny.

Lidén's (1986) classification was in a relatively early stage and only involved 19 sections to accommodate the 250–300 *Corydalis* species known in that age, which was far from enough to ensure a well-defined classification for such a species-rich genus. Moreover, Lidén has contributed and approved the classification of *Corydalis* into 40 sections and five series in Zhang et al. (2008) treatment. Thus, we did not compare Lidén's (1986) classification in detail with our phylogeny.

**Figure 5:**
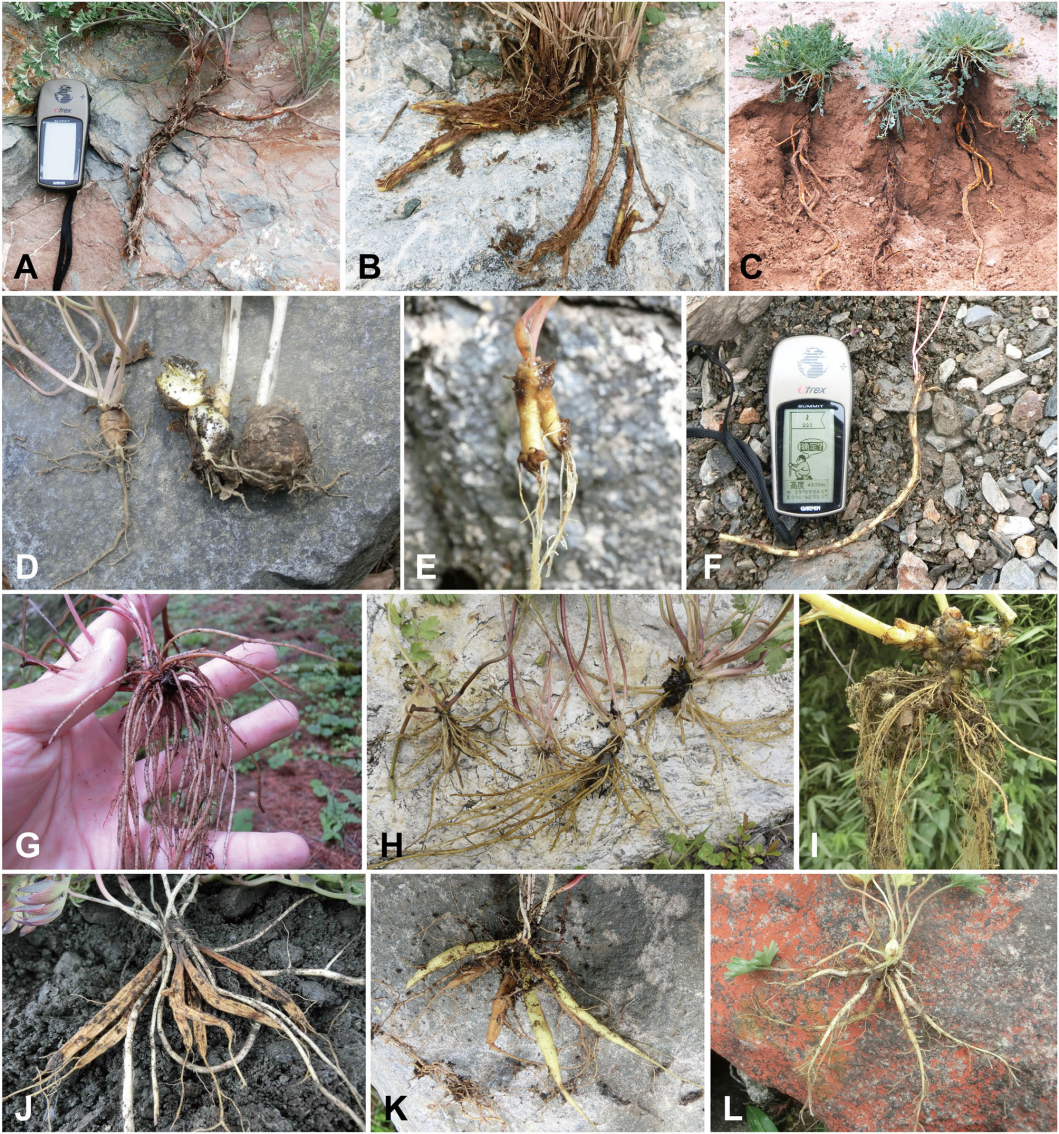
Morphology of root and rhizome of *Corydalis* species. With taproot: **(A)** *C. stricta*, clade I; **(B)** *C. livida*, clade IV; **(C)** *C. wuzhengyiana*, clade V. With tuber; **(D)** *C. caudata*, clade IV; **(E)** *C. hsiaowutaishanensis*, clade IV. With long thin rootstock; **(F)** *C. retingensis*, clade IV. With fibrous root; **(G)** *Corydalis* sp. (sect. *Davidianae*), clade V; **(H)** *C. elata*, clade VI; **(I)** *C. mucronate*, clade VI. With enlarged storage root; **(J)** *C. trachycarpa*, clade V; **(K)** *C. minutiflora*, clade VI; **(L)** *C. pseudoadoxa*, clade VI.

Wu et al. (1996, 1999) took into consideration the gross morphological characters, habitats, and distributions and summarized them into 83 evolutionary routes. According to those evolutionary routes, they divided *Corydalis* into two groups (*Corydalis* and *Pistolochia*) and 40 sections and further deduced the possible origin and evolution of the sections. Wu et al. (1996, 1999) initially emphasized the underground organs (Figure 5) to arrange the sections and thought of the fibrous root sect. *Asterostigma* as the most primitive, while the tuberous sections were relatively evolved. If we number and order the sections in our plastome phylogeny (in Figure 1) from the bottom to the top as I(1)-II(1-2-3-4)-III(1)-IV(1-2-3-4-5-6)-V(1-2-3-4-5-6-7-8-9-10)-VI(1-2-3-4-5-6-7-8-9-10-11-12-13), then Wu et al's (1996, 1999) classification system is as below, *Corydalis* group (VI3-VI8-?-III1-VI5-V1-VI7-?-V4-VI10-^*^-?-V9-IV2-?-V10-V6-V7-?-^*^-V3-?-IV5-?-#VI4-IV4-VI4-II4-VI1-?-I1-#I1-II3-II1-II2)-*Pistolochia* group (IV1-IV6-IV3-?-?) (“?” represent a section that we have no material; “^*^” represent a section that has been transferred to more than one sections; “#” indicate a section has been transferred to the following section). We found that Wu et al.'s classification differed remarkably with our phylogeny. The sections that belong to the same monophyletic clade in our molecular phylogeny were scattered in Wu et al.'s sectional arrangement. None of the two groups in Wu et al's (1996, 1999) classification is monophyletic. The fibrous root sections in clades V and VI, which were thought to be most primitive (Wu et al., 1996, 1999), were relatively posteriorly diverged in *Corydalis*.

Zhang et al.'s (2008) classification system partially adopted the results of previous molecular phylogenetic researches and divided *Corydalis* into three subgenera. However, owing to the lack of enough molecular data in subg. *Corydalis* s. l., the relationships within this subgenus were not well resolved. Following the coding of sections in the previous paragraph, Zhang et al.'s (2008) classification system is as below, Subg. *Cremnocapnos* (?-I1) - subg. *Sophorocapnos* (II3-II1-II4-II2) - subg. *Corydalis* s. l. (IV6-?-IV4-IV3-?-?-IV5-IV2-?-IV1-III1-V1-?-V5-?-V8-?-V7-V3-V9-V10- V6-VI3-?-V4-?-VI10-VI12-VI11-VI13-VI8-VI5-VI9-VI4-VI6-VI7-?-VI1-VI2) (“?” represent a section that we have no material). The sectional arrangement of Zhang et al.'s classification was more rational. The first two subgenera are congruent with our molecular phylogeny. The most controversies exist in the largest subg. *Corydalis* s. l., which was the combination of sections from clades III, IV, V, and VI. The sections belonging to the same clade in our molecular phylogeny were also scattered in Zhang et al.'s subg. *Corydalis*. For example, because sharing high levels of homoplasy in enlarged fascicled storage root (Figures 5J–L), sect. *Trachycarpae* and the four series (ser. *Curviflorae*, ser. *Kokianae*, ser. *Fusiformes*, and ser. *Clavatae*) were closely arranged in Zhang et al.'s classification, but these taxa belong to different clades. Some extensive and in-depth research are needed to address the gap between morphological similarity and molecular phylogeny mismatches.

Although, we could morphologically confirm the affiliation of one species to a section according to the latest classification system of *Corydalis* (Zhang et al., 2008), a possibility still exists as to the sections being non-monophyletic. Some detailed phylogenetic studies, that focused on a specific section, are needed to extend our understanding about the relationships within *Corydalis* sections and probably revise the relationship between a few sections as well.

### Classification of the Tuberous *Corydalis* Species

Tuber is an important character of some *Corydalis* species. This character has been emphasized before by some authors to classify the tuberous species into a subgenus (subg. *Capnites* de Candolle, 1821; Popov, 1937; Su and Wu, 1985) or a group (*Pistolochia* group; Wu et al., 1996, 1999). Subgenus *Capnites* or *Pistolochia* group, although differed conceptually, were the same assemblage of species. Here, we took Wu et al's (1999) treatment as an example to discuss the conflicts between the morphology-based and our phylogeny-based classification of the tuberous species. Wu et al's (1999) *Pistolochia* group was composed of five sections, i.e., sect. *Dactylotuber*, sect. *Duplotuber*, sect. *Pesgallinaceus* (sect. *Corydalis* in Zhang et al., 2008), sect. *Leonticoides*, and sect. *Radixcava*. In this study, we included representatives of the former three sections, but they turned out to be polyphyletic and not a natural group. The oblong tuber section *Dactylotuber* (*C. hsiaowutaishanensis*, Figure 5E) and long thin fleshy rootstock sect. *Oocapnos* (*C. retingensis*, Figure 5F) formed a monophyletic clade (PP = 1, BS = 100), the rounded tuber sect. *Pesgallinaceus* (sect. *Corydalis* in Zhang et al., 2008; *C. caudata*, Figure 5D) and oblong tuber sect. *Benecinctae* (*C. benecincta*) formed another monophyletic clade (PP = 1, BS = 100), the rounded tuber section *Duplotuber* (*C. decumbens*) and taproot sect. *Flaccidae* (*C. livida*, Figure 5B) formed the last monophyletic clade (PP = 1, BS = 94) within clade IV. The three tuberous and three non-tuberous sections, which, in total, displayed four types of roots and rhizomes, i.e., oblong tuber, rounded tuber, long thin fleshy rootstock, and taproot, grouped in a monophyletic clade IV. Thus, the classification of some researchers (de Candolle, 1821; Popov, 1937; Su and Wu, 1985; Wu et al., 1996, 1999) to treat the tuberous species in a separate subgenus or group is not supported. As we did not have materials for another two tuberous sections (sect. *Leonticoides* and sect. *Radixcava*) and three non-tuberous sections (sect. *Capnogorium*, sect. *Kingianae*, and sect. *Rupifragae*; Zhang et al., 2008) belonging to clade IV, a more extensive research is needed to completely resolve the phylogenetic relationships between the tuberous and non-tuberous sections within this clade.

### Convergent and Divergent Evolution of Root and Rhizome in *Corydalis*

Root is one of the most important organs for plants, which is responsible for the absorption of water and mineral elements. In the long evolutionary history of *Corydalis*, different types of roots and associated structures (such as rhizome) have evolved (Figure 5) to better adapt to the various environments. The taxonomy of *Corydalis* has been largely based on the morphology of root and rhizome, such as the classification of *Corydalis* into tuberous or non-tuberous taxa (though it was incorrect), and the division of sect. *Rapiferae* (in Wu et al., 1996, 1999) into five series (Zhang et al., 2008). However, according to the phylogram in this study, the root and rhizome of *Corydalis* have undergone multiple independent divergent and convergent evolutions.

Some *Corydalis* species, though sharing the same ancestor, when adapted to the diverse environment, have evolved with markedly different roots and rhizomes. The species in clade IV, though belonging to the same clade, have displayed four types of root and rhizome morphologies (Figures 5B,D–F). The tuberous species are often distributed in the understory, while the oblong tuber and long thin fleshy rootstock species often grow in the stony scree, and the taproot species are often distributed in the xeric habitat. For the species in clade V, although the majority were characterized with taproot, fibrous root has originated at least two times in this clade: one in the first diverged sect. *Davidianae* and the other in sect. *Trachycarpae*. Clade VI species were mostly characterized by the fibrous root, while the first diverged sect. *Chinenses* and sect. *Vermiculares* were unique with a taproot.

On the other hand, convergent evolutions were also frequently observed within *Corydalis*. The species that are from different clades, when grown in the same or similar environment, can evolve to share similar root characteristics. In clade I, sect. *Strictae* species adapts to the central Asia xeric habitat; in clade II, sect. *Thalictrifoliae* species adapts to the dry cliff habitat; in clade IV, sect. *Flaccidae* species adapts to the northwest China xeric habitat; and in clade V, sect. *Chrysocapnos*, sect. *Latiflorae*, and sect. *Mucroniferae* species adapt to the xeric habitat in the Qinghai-Tibet Plateau, all shared a similar long taproot (Figures 5A–C). The species from sect. *Trachycarpae* (clade V) and the four series (ser. *Curviflorae*, ser. *Kokianae*, ser. *Fusiformes*, and ser. *Clavatae*; clade VI) are all adapted to the grasslands, meadows, or scree environment and share similarly enlarged fascicled storage roots (Figures 5J–L).

Different types of roots and rhizomes were scattered in the molecular phylogeny, indicating that they may not be suitable to distinguish different sections within *Corydalis* and should be used with caution in the taxonomy of *Corydalis*. Multiple genomic and transcriptomic data should be combined in the future to completely illustrate the convergent and divergent evolution of root and rhizome within *Corydalis*.

### Origin and Divergence of *Corydalis*

Little is known about the origin and divergence of *Corydalis*, due to the lack of informative fossils. The few fossil records of *Corydalis* from the uppermost Miocene in Germany (Collinson et al., 1993) and from late Pliocene in the Hengduan Mountain region of China (Huang et al., 2021) were probably too young to deduce the origin of *Corydalis*. As an alternative, we estimated the origin and divergence of *Corydalis* following a two-step molecular dating analysis. In this study, the subfamily Fumarioideae, where *Corydalis* belongs, was estimated to have originated in 92.81 Ma (stem age, 95% HPD: 117.06–32.90) in the early Upper Cretaceous. This age was approximate to the estimation of 96 Ma by Xu et al. (2022) using 78 single-copy genes from *Corydalis tomentella*, which mutually corroborated that our estimations were rational. In our estimation, the *Corydalis* ancestor split from *Lamprocapnos* about 65.65 Ma (95% HPD: 67.60–63.72) at the beginning of Cenozoic and start to diversify in early Eocene (crown age 49.08 Ma, 95% HPD: 51.03–47.18). We are still not clear of the timeline when the most recent *Corydalis* ancestor has emerged, due to the lack of materials for some other genera that diverged right before *Corydalis* (*Dicentra, Ichtyoselmis, Dactylicapnos*, etc.; Pérez-Gutiérrez et al., 2015; Sauquet et al., 2015). Within *Corydalis*, the time interval for lineage evolution differed significantly. The first *Corydalis* clade (I) diverged ca. 49.08 Ma, and it took almost 16 million years for the second and third *Corydalis* clades to come into being, while it only took ca. 5 million years for the last three clades. It may be interesting to elucidate the mechanisms behind this heterogeneity. Since around 25.49 Ma, the species-rich clades (clade II, 22.29 Ma; IV, 25.49 Ma; V, 22.32 Ma; VI, 22.43 Ma), especially clades V and VI, radiated almost simultaneously. Clade II species are mostly distributed in the east part of East Asia, while species from the latter three clades were predominantly distributed on the Qinghai-Tibet Plateau (QTP). The uplift of the Qinghai-Tibet Plateau (QTP) from 25 Ma to 17 Ma has changed the environment of East Asia dramatically (Shi et al., 1999), which justly overlapped and has probably triggered the above radiation of *Corydalis* species. Clades V and VI, the two most species-rich clades in *Corydalis*, may represent ideal taxa to finely elucidate how the uplift of QTP has triggered the evolution of species in this region in future research. The two species in clade III, despite belonging to the same section (sect. *Archaeocapnos*), diverged 26.61 Ma (95% HPD: 34.97–17.86), which was much older than the divergence time between sections within the other clades. It remains to be determined whether it is still proper to keep those species in a single section. In this study, we just offered a rough framework for the origin and divergence of *Corydalis*, and a larger sampling is still needed to provide further detailed information on the divergence of *Corydalis* species.

## Conclusion

In this study, by using plastome data from the species representing ca. 80% of the sections and series of *Corydalis*, we presented the first reliable, highly resolved, and well-supported backbone phylogeny for *Corydalis* at the sectional level. The robust phylogeny obtained in this study offers new insights into the systematic relationships among the sections and series of *Corydalis* and will serve as a framework for upcoming research on the classification, evolution, and biogeography of *Corydalis*. However, uncertainty and curiosity remain for the unsampled sections and species. An extended sampling plan to cover more *Corydalis* species is needed to better complete the Tree of Life for *Corydalis*.

## Data Availability Statement

The datasets presented in this study can be found in online repositories. The names of the repository/repositories and accession number(s) can be found below: https://www.ncbi.nlm.nih.gov/genbank/, ON152770-ON152801.

## Author Contributions

DW and XX conceived the study and collected the materials. XX performed analyses and drafted the manuscript. DW provided suggestions on structuring the article and the main points of the discussion and revised the manuscript. XL helped with analyses. All authors read and approved the final manuscript.

## Funding

This work was supported by grants from the National Natural Science Foundation of China (31170310), the Science and Technology Basic Work (2013FY112100 and 2013FY112300), the Special Foundation for the Specimen Platform of China, Teaching Specimen Sub-Platform, web, http://mnh.scu.edu.cn/ (2005DKA21403-JK), the Fundamental Research Funds for the Central Universities (Excellent doctoral dissertation development program of CCNU, 202050185085), and funds from the Shaanxi Provincial Bio-Resource Key Laboratory, Shaanxi University of Technology (SLGPT2019KF03-02).

## Conflict of Interest

The authors declare that the research was conducted in the absence of any commercial or financial relationships that could be construed as a potential conflict of interest.

## Publisher's Note

All claims expressed in this article are solely those of the authors and do not necessarily represent those of their affiliated organizations, or those of the publisher, the editors and the reviewers. Any product that may be evaluated in this article, or claim that may be made by its manufacturer, is not guaranteed or endorsed by the publisher.

## Supplementary Material

Supplementary Figure 1 The initial chronogram shows the divergence time of *Corydalis* estimated in BEAST based on plastomes data from six representative *Corydalis* and 21 outgroup species. Estimated ages are shown near the nodes, and blue bars represent 95% high posterior density. Calibration points (Node 1 and Node 2) were indicated with a black solid circle.

Supplementary Table 1 Detailed information for plastome sequencing and assembling results. The column “Assembling result” indicates the numbers of scaffolds that belong to the corresponding part, LSC, IR, and SSC, respectively. For “Total length”, the length of IR was counted two times in the uncircularized plastome.

Supplementary Table 2 Optimal partitioning scheme, best-fit substitution models, and related parameters for the BI analysis. The sign after a gene, such as “-1,” “-2,” and “-3,” indicate the nucleotide that corresponds to the first, second, or third positions of the codon, respectively.

Supplementary Table 3 Optimal partitioning scheme and related parameters using GTR+I+G substitution model for the ML analysis. The sign after a gene, such as “-1,” “-2,” or “-3,” indicates the nucleotide that corresponds to the first, second, or third positions of the codon, respectively.

Supplementary Table 4 Ages of *Corydalis* clades estimated using BEAST. Age represents stem age (S) or the age of split from most recent common ancestors, unless otherwise indicated (crown age, C).

## References

[B1] Alhassen L. Dabbous T. Ha A. Dang L. H. L. Civelli O. (2021). The analgesic properties of *Corydalis yanhusuo*. Molecules 26, 7498. 10.3390/molecules26247498 34946576 PMC8704877

[B2] Barrett C. F. Baker W. J. Comer J. R. Conran J. G. Lahmeyer S. C. LeebensMack J. H.. (2016). Plastid genomes reveal support for deep phylogenetic relationships and extensive rate variation among palms and other commelinid monocots. New Phytol. 209, 855–870. 10.1111/nph.13617 26350789

[B3] Bouckaert R. Vaughan T. G. Barido-Sottani J. Duchêne S. Fourment M. Gavryushkina A.. (2019). BEAST 2.5: an advanced software platform for Bayesian evolutionary analysis. PLoS Comput. Biol. 15, e1006650. 10.1371/journal.pcbi.1006650 30958812 PMC6472827

[B4] Capella-Gutiérrez S. Silla-Martínez J. M. Gabaldón T. (2009). trimAl: a tool for automated alignment trimming in large-scale phylogenetic analyses. Bioinformatics 25, 1972–1973. 10.1093/bioinformatics/btp348 19505945 PMC2712344

[B5] Catalogue of Life (2022). Corydalis DC. Available online at: https://www.catalogueoflife.org/data/taxon/62LZP (accessed March 4, 2022).

[B6] Chinese Pharmacopoeia Commission (2015). Pharmacopoeia of the People's Republic of China, Volume 1. Beijing: China Medical Science Press.

[B7] Chlebek J. Macakova K. Cahlikova L. Kurfurst M. Kunes J. Opletal L. (2011). Acetylcholinesterase and butyrylcholinesterase inhibitory compounds from *Corydalis Cava* (Fumariaceae). Nat. Prod. Commun. 6, 607–610. 10.1177/1934578X1100600507 21615017

[B8] Clegg M. T. Gaut B. S. Learn G. H. Jr. Morton B. R. (1994). Rates and patterns of chloroplast DNA evolution. Proc. Natl. Acad. Sci. USA. 91, 6795–6801. 10.1073/pnas.91.15.6795 8041699 PMC44285

[B9] Cock P. J. A. Antao T. Chang J. T. Chapman B. A. Cox C. J. Dalke A.. (2009). Biopython: freely available Python tools for computational molecular biology and bioinformatics. Bioinformatics. 25, 1422–1423. 10.1093/bioinformatics/btp163 19304878 PMC2682512

[B10] Collinson M. E. Boulter M. C. Holmes P. L. (1993). Magnoliophyta ('Angiospermae'), in The Fossil Record 2., ed Benton M. J. (London: Chapman and Hall), 809–841.

[B11] Cosner M. E. Raubeson L. A. Jansen R. K. (2004). Chloroplast DNA rearrangements in Campanulaceae: phylogenetic utility of highly rearranged genomes. BMC Evol. Biol. 4, 27. 10.1186/1471-2148-4-27 15324459 PMC516026

[B12] Darling A. C. E. Mau B. Blattner F. R. Perna N. T. (2004). Mauve: multiple alignment of conserved genomic sequence with rearrangements. Genome Res. 14, 1394–1403. 10.1101/gr.2289704 15231754 PMC442156

[B13] Darriba D. Taboada G. L. Doallo R. Posada D. (2012). jModelTest 2: more models, new heuristics and parallel computing. Nat. Methods 9, 772. 10.1038/nmeth.2109 22847109 PMC4594756

[B14] de Candolle A. P. (1805). Papaveraceae, in Flore francaise, 3rd, vol. 4, ed Lamarck De Candolle (Paris: Chez H. Agasse), 636–638.

[B15] de Candolle A. P. (1821). Regni vegetabilis systema naturale, sive Ordines, genera et species plantarum secundum methodi naturalis normas digestarum et descriptarum [Syst. Nat.], Volume v.2. Parisiis: Sumptibus sociorum Treuttel et Würtz.

[B16] Deng A. P. Zhang Y. Zhou L. Kang C. Z. Lv C. G. Kang L. P.. (2021). Systematic review of the alkaloid constituents in several important medicinal plants of the Genus *Corydalis*. Phytochemistry 183, 112644. 10.1016/j.phytochem.2020.112644 33429352

[B17] Downie S. R. Palmer J. D. (1992). Use of chloroplast DNA rearrangements in reconstructing plant phylogeny, in Plant Molecular Systematics, eds Soltis P. Soltis D. Doyle J. J. (New York: Chapman and Hall), 14–35. 10.1007/978-1-4615-3276-7_2

[B18] Doyle J. A. Hotton C. L. (1991). Diversification of early angiosperm pollen in a cladistic context, in Pollen and Spores: Patterns of Diversification, eds Blackmore S. Barnes S. H. (Oxford: Clarendon Press), 169–195.

[B19] Doyle J. J. Doyle J. L. Ballenger J. A. Palmer J. D. (1996). The distribution and phylogenetic significance of a 50-kb chloroplast DNA inversion in the flowering plant family leguminosae. Mol. Phylogenet. Evol. 5, 429–438. 10.1006/mpev.1996.0038 8728401

[B20] Editorial Board of Chinese Tibetan medicine (1996). Chinese Tibetan Medicine. Shanghai: Shanghai Science and Technology Press.

[B21] Ehlers B. K. Olesen J. M. (2004). Flower production in relation to individual plant age and leaf production among different patches of *Corydalis intermedia*. Plant Ecol. 174, 71–78. 10.1023/B:VEGE.0000046060.77491.b9

[B22] Fedde F. (1924). Neue Arten von *Corydalis* aus China, VI. Repert. Spec. Nov. Regni Veg. 20, 50–63. 10.1002/fedr.19240200109

[B23] Fedde F. (1926). Neue Arten von *Corydalis* aus China, ?. Repert. Spec. Nov. Regni Veg. 23, 180–182. 10.1002/fedr.19260231204

[B24] Fedde F. (1936). Papaveraceae, in Die natuerlichen Pflanzenfamilien, 2nd, eds Engler A. Prantl K. (Leipzig: W. Engelmann), 5–145.

[B25] Greiner S. Lehwark P. Bock R. (2019). OrganellarGenomeDRAW (OGDRAW) version 1.3.1: expanded toolkit for the graphical visualization of organellar genomes. Nucl. Acids Res. 47, W59–W64. 10.1093/nar/gkz238 30949694 PMC6602502

[B26] Hansen D. R. Dastidar S. G. Cai Z. Penaflor C. Kuehl J. V. Boore J. L.. (2007). Phylogenetic and evolutionary implications of complete chloroplast genome sequences of four early-diverging angiosperms: *Buxus* (Buxaceae), *Chloranthus* (Chloranthaceae), *Dioscorea* (Dioscoreaceae), and *Illicium* (Schisandraceae). Mol. Phylogenet. Evol. 45, 547–563. 10.1016/j.ympev.2007.06.004 17644003

[B27] Huang X. M. Xu X. D. Wang D. (2022). Insight from newly sequenced chloroplast genome challenges the primitive position of *Corydalis temulifolia* (Papaveraceae). Phytotaxa 548: 223–239. 10.11646/phytotaxa.548.2.6

[B28] Huang Y. J. Zhu H. Su T. Spicer R. A. Hu J. J. Jia L. B.. (2021). The rise of herbaceous diversity at southeastern margin of the Tibetan Plateau: first insight from fossils. J. Syst. Evol. 1–15. 10.1111/jse.12755

[B29] Hughes N. F. (1994). The Enigma of Angiosperm Origins. Cambridge: Cambridge University Press.

[B30] Hughes N. F. McDougall A. B. (1990). Barremian-Aptian angiospermid pollen records from southern England. Rev. Palaeobot. Palyno. 65, 145–151. 10.1016/0034-6667(90)90065-Q

[B31] Jansen R. K. Cai Z. Raubeson L. A. Daniell H. Leebens-Mack J. Müller K. F.. (2007). Analysis of 81 genes from 64 plastid genomes resolves relationships in angiosperms and identifies genome-scale evolutionary patterns. Proc. Natl. Acad. Sci. USA 104, 19369–19374. 10.1073/pnas.0709121104 18048330 PMC2148296

[B32] Jansen R. K. Palmer J. D. (1987). A chloroplast DNA inversion marks an ancient evolutionary split in the sunflower family (Asteraceae). Proc. Natl. Acad. Sci. USA. 84, 5818–5822. 10.1073/pnas.84.16.5818 16593871 PMC298954

[B33] Jansen R. K. Ruhlman T. A. (2012). Plastid genomes of seed plants, in Genomics of Chloroplasts and Mitochondria, advances in photosynthesis and respiration, Vol. 35, eds Bock R. Knoop V. (Dordrecht: Springer Netherlands), 103–126. 10.1007/978-94-007-2920-9_5

[B34] Jiang L. Li M. H. Zhao F. X. Chu S. S. Zhang L. P. Xu T.. (2018). Molecular identification and taxonomic implication of herbal species in genus *Corydalis* (Papaveraceae). Molecules 23, 1–10. 10.3390/molecules23061393 29890665 PMC6100380

[B35] Kanwal N. Zhang X. Afzal N. Yang J. Li Z. H. Zhao G. F. (2019). Complete chloroplast genome of a Chinese endemic species *Corydalis trisecta* Franch. (Papaveraceae). Mitochondrial DNA B Resour. 4, 2291–2292. 10.1080/23802359.2019.1627930 33365510 PMC7687375

[B36] Katoh K. Standley D. M. (2013). MAFFT multiple sequence alignment software version 7: improvements in performance and usability. Mol. Biol. Evol. 30, 772–780. 10.1093/molbev/mst010 23329690 PMC3603318

[B37] Kim H. W. Kim K. J. (2016). Complete plastid genome sequences of *Coreanomecon hylomeconoides* Nakai (Papaveraceae), a Korea endemic genus. Mitochondrial DNA B Resour. 1, 601–602. 10.1080/23802359.2016.1209089 33473566 PMC7800022

[B38] Kim S. R. Hwang S. Y. Jang Y. P. Park M. J. Markelonis G. J. Oh T. H.. (1999). Protopine from *Corydalis ternata* has anticholinesterase and antiamnesic activities. Planta Med. 65, 218–221. 10.1055/s-1999-13983 10232064

[B39] Kudo G. Maeda T. Narita K. (2001). Variation in floral sex allocation and reproductive success within inflorescences of *Corydalis ambigua* (Fumariaceae) pollination efficiency or resource limitation. J. Ecol. 89, 48–56. 10.1046/j.1365-2745.2001.00512.x

[B40] Li B. Li Y. D. Cai Q. F. Lin F. R. Huang P. Zheng Y. Q. (2016). Development of chloroplast genomic resources for *Akebia quinata* (Lardizabalaceae). Conserv. Genet. Resour. 8, 447–449. 10.1007/s12686-016-0593-0

[B41] Li H. T. Yi T. S. Gao L. M. Ma P. F. Zhang T. Yang J. B.. (2019). Origin of angiosperms and the puzzle of the Jurassic gap. Nat. Plants 5, 461–470. 10.1038/s41477-019-0421-0 31061536

[B42] Li T. J. Fu X. C. Deng H. S. Han X. J. Wen F. Xu L. L. (2019). The complete chloroplast genome of *Ranunculus Cantoniensis*. Mitochondrial DNA B Resour. 4, 1095–1096. 10.1080/23802359.2019.1586483

[B43] Lidén M. (1986). Synopsis of Fumarioideae with a monograph of the tribe Fumarieae. Opera Bot. 88, 1–133.

[B44] Lidén M. Fukuhara T. Axberg T. (1995). Phylogeny of *Corydalis*, ITS and morphology. Plant Syst. Evol. 9, 183–188. 10.1007/978-3-7091-6612-3_17

[B45] Lidén M. Fukuhara T. Rylander J. Oxelman B. (1997). Phylogeny and classification of Fumariaceae, with emphasis on *Dicentra* s. l., based on the chloroplast gene rps16 intron. Plant Syst. Evol. 206, 411–420. 10.1007/BF00987960

[B46] Liu Y. Y. Kan S. L. Wang J. L. Cao Y. N. Li J. M.. (2021). Complete chloroplast genome sequences of *Corydalis edulis* and *Corydalis shensiana* (Papaveraceae). Mitochondrial DNA B Resour. 6, 257–258. 10.1080/23802359.2020.1863167 33659648 PMC7872545

[B47] Luo D. S. Feng C. H. Xia G. C. (1984). The resources of the Tibetan drugs in Qinghai-Xizang Plateau: preliminary studies on the plants of *Corydalis*. Zhong Cao Yao 15, 33–36.

[B48] Lynch R. C. Kane N. C. (2014). Vitis Rotundifolia Chloroplast, Complete Genome. Available online at: https://www.ncbi.nlm.nih.gov/nuccore/NC_023790 (accessed March 4, 2022).

[B49] Ma J. Yang B. Zhu W. Sun L. Tian J. Wang X. (2013). The complete chloroplast genome sequence of *Mahonia bealei* (Berberidaceae) reveals a significant expansion of the inverted repeat and phylogenetic relationship with other angiosperms. Gene 528, 120–131. 10.1016/j.gene.2013.07.037 23900198

[B50] Ma P. F. Zhang Y. X. Zeng C. X. Guo Z. H. Li D. Z. (2014). Chloroplast phylogenomic analyses resolve deep-level relationships of an intractable bamboo tribe Arundinarieae (Poaceae). Syst. Biol. 63, 933–950. 10.1093/sysbio/syu054 25092479

[B51] Magallón S. Gómez-Acevedo S. Sánchez-Reyes L. L. Hernández-Hernández T. (2015). A metacalibrated time-tree documents the early rise of flowering plant phylogenetic diversity. New Phytol. 207, 437–453. 10.1111/nph.13264 25615647

[B52] Moore M. J. Bell C. D. Soltis P. S. Soltis D. E. (2007). Using plastid genome-scale data to resolve enigmatic relationships among basal angiosperms. Proc. Natl. Acad. Sci. U. S. A. 104, 19363–19368. 10.1073/pnas.0708072104 18048334 PMC2148295

[B53] Moore M. J. Dhingra A. Soltis P. S. Shaw R. Farmerie W. G. Folta K. M.. (2006). Rapid and accurate pyrosequencing of angiosperm plastid genomes. BMC Plant Biol. 6, 17. 10.1186/1471-2229-6-17 16934154 PMC1564139

[B54] Moore M. J. Soltis P. S. Bell C. D. Burleigh J. G. Soltis D. E. (2010). Phylogenetic analysis of 83 plastid genes further resolves the early diversification of eudicots. Proc. Natl. Acad. Sci. U.S.A. 107, 4623–4628. 10.1073/pnas.0907801107 20176954 PMC2842043

[B55] Mower J. P. Vickrey T. L. (2018). Structural diversity among plastid genomes of land plants. Adv. Bot. Res. 85, 263–292. 10.1016/bs.abr.2017.11.013

[B56] Niu Y. Chen G. Peng D. L. Song B. Yang Y. Li Z. M.. (2014). Grey leaves in an alpine plant: a cryptic colouration to avoid attack. New Phytol. 203, 953–963. 10.1111/nph.12834 24800901

[B57] Niu Y. Chen Z. Stevens M. Sun H. (2017). Divergence in cryptic leaf colour provides local camouflage in an alpine plant. Proc. R. Soc. B 284, 20171654. 10.1098/rspb.2017.1654 28978734 PMC5647307

[B58] Ohara M. Higashi S. (1994). Effects of inflorescence size on visits from pollinators and seed set of *Corydalis ambigua* (Papaveraceae). Oecologia 98, 25–30. 10.1007/BF00326086 28312792

[B59] Ohkawara K. Ohara M. Higashi S. (1997). The evolution of ant-dispersal in a spring-ephemeral *Corydalis ambigua* (Papaveraceae): timing of seed-fall and effects of ants and ground beetles. Ecography 20, 217–223. 10.1111/j.1600-0587.1997.tb00364.x

[B60] Palmer J. D. (1985). Comparative organization of chloroplast genomes. Annu. Rev. Genet. 19, 325–354. 10.1146/annurev.ge.19.120185.001545 3936406

[B61] Park S. An B. Park S. (2018). Reconfiguration of the plastid genome in *Lamprocapnos spectabilis*: IR boundary shifting, inversion, and intraspecific variation. Sci. Rep. 8, 13568. 10.1038/s41598-018-31938-w 30206286 PMC6134119

[B62] Pérez-Gutiérrez M. A. Romero-García A. T. Fernández M. C. Blanca G. Salinas-Bonillo M. J. Suárez-Santiago V. N. (2015). Evolutionary history of fumitories (subfamily Fumarioideae, Papaveraceae): an old story shaped by the main geological and climatic events in the northern hemisphere. Mol. Phylogenet. Evol. 88, 75–92. 10.1016/j.ympev.2015.03.026 25862377

[B63] Persoon C. H. (1806). Synopsis Plantarum, vol. 2. Parisiis Lutetiorum: C.F. Cramerum.

[B64] Popov M. (1937). Corydalis, in Flora URSS, vol. VII, ed Komarov V. L. (Moscow: Leningrad), 649–706.

[B65] Qu X. J. Moore M. J. Li D. Z. Yi T. S. (2019). PGA: a software package for rapid, accurate, and fexible batch annotation of plastomes. Plant Methods. 15, 50. 10.1186/s13007-019-0435-7 31139240 PMC6528300

[B66] Rambaut A. (2018). FigTree v1.4.4. Available online at: http://tree.bio.ed.ac.uk/software/figtree/ (accessed April 21, 2020).

[B67] Rambaut A. Drummond A. J. Xie D. Baele G. Suchard M. A. (2018). Posterior summarisation in Bayesian phylogenetics using Tracer 1.7. Syst. Biol. 67, 901–904. 10.1093/sysbio/syy032 29718447 PMC6101584

[B68] Ren F. M. Wang L. Q. Li Y. Zhuo W. Xu Z. C. Guo H. J.. (2021). Highly variable chloroplast genome from two endangered Papaveraceae lithophytes *Corydalis tomentella* and *Corydalis saxicola*. Ecol. Evol. 11, 4158–4171. 10.1002/ece3.7312 33976800 PMC8093665

[B69] Ren F. M. Wang Y. W. Xu Z. C. Li Y. Xin T. Y. Zhou J. G.. (2018). DNA barcoding of *Corydalis*, the most taxonomically complicated genus of Papaveraceae. Ecol. Evol. 9, 1934–1945. 10.1002/ece3.4886 30847083 PMC6392370

[B70] Ronquist F. Teslenk M. Van der Mark P. Ayres D. Darling A. Höhna S.. (2012). MrBayes 3.2: efficient Bayesian phylogenetic inference and model choice across a large model space. Syst. Biol. 61, 539–542. 10.1093/sysbio/sys029 22357727 PMC3329765

[B71] Ruhlman T. A. Jansen R. K. (2014). The plastid genomes of flowering plants, in Chloroplast Biotechnology: Methods and Protocols, ed Maliga P. (New York: Springer), 3–38. 10.1007/978-1-62703-995-6_1

[B72] Sauquet H. Carrive L. Poullain N. Sannier J. Damerval C. Nadot S. (2015). Zygomorphy evolved from disymmetry in Fumarioideae (Papaveraceae, Ranunculales): new evidence from an expanded molecular phylogenetic framework. Ann. Bot. 115, 895–914. 10.1093/aob/mcv020 25814061 PMC4407061

[B73] Shi L. C. Chen H. M. Jiang M. Wang L. Q. Wu X. Huang L. F.. (2019). CPGAVAS2, an integrated plastome sequence annotator and analyzer. Nucl. Acids Res. 47, W65–W73. 10.1093/nar/gkz345 31066451 PMC6602467

[B74] Shi Y. F. Li J. J. Li B. Y. Yao T. D. Wang S. M. Li S. J.. (1999). Uplift of the Qinghai—Xizang (Tibetan) plateau and East Asia environmental change during late Cenozoic. Acta Geogr. Sin. 54, 10–20.

[B75] Stamatakis A. (2014). RAxML version 8: a tool for phylogenetic analysis and post-analysis of large phylogenies. Bioinformatics 30, 1312–1313. 10.1093/bioinformatics/btu033 24451623 PMC3998144

[B76] Su Z. Y. (1980). The classification and distribution of sect. Mucroniferae Fedde of Corydalis Vent. in China. Acta Bot. Yunnan. 2, 202–212.

[B77] Su Z. Y. Wu C. Y. (1985). A study on the classification, distribution, evolutionary trends and uses of Chinese *Corydalis* subgen. *Capnites* DC. Acta Bot. Yunnan. 7, 253–276.

[B78] Sun Y. X. Moore M. J. Lin N. Adelalu K. F. Meng A. Jian S.. (2017). Complete plastome sequencing of both living species of Circaeasteraceae (Ranunculales) reveals unusual rearrangements and the loss of the ndh gene family. BMC Genom. 18, 592. 10.1186/s12864-017-3956-3 28793854 PMC5551029

[B79] Sun Y. X. Moore M. J. Meng A. Soltis P. S. Soltis D. E. Li J.. (2013). Complete plastid genome sequencing of trochodendraceae reveals a significant expansion of the inverted repeat and suggests a paleogene divergence between the two extant species. PLoS ONE 8, e60429. 10.1371/journal.pone.0060429 23577110 PMC3618518

[B80] Sun Y. X. Moore M. J. Zhang S. J. Soltis P. S. Soltis D. E. Zhao T. T.. (2016). Phylogenomic and structural analyses of 18 complete plastomes across nearly all families of early-diverging eudicots, including an angiosperm-wide analysis of IR gene content evolution. Mol. Phylogenet. Evol. 96, 93–101. 10.1016/j.ympev.2015.12.006 26724406

[B81] von Balthazar M. Pedersen K. Friis E. (2005). *Teixeiraea lusitanica*, a new fossil flower from the early cretaceous of portugal with affinities to the ranunculales. Plant Syst. Evol. 255, 55–75. 10.1007/s00606-005-0347-z

[B82] Wang Q. Lei Z. X. Zhou L. R. Mai B. W. Zhu N. Y. Zhao X. L.. (2021). Characterization of the complete chloroplast genome of *Corydalis bungeana* Turcz. Mitochondrial DNA B Resour. 6, 1971–1972. 10.1080/23802359.2021.1925984 34179484 PMC8204951

[B83] Wang Y. W. (2006). Systematics of Corydalis DC. (Fumariaceae). PhD Thesis. Beijing: Institute of Botany, The Chinese Academy of Sciences.

[B84] Wicke S. Schneeweiss G. M. dePamphilis C. W. Müller K. F. Quandt D. (2011). The evolution of the plastid chromosome in land plants: gene content, gene order, gene function. Plant Mol. Biol. 76, 273–297. 10.1007/s11103-011-9762-4 21424877 PMC3104136

[B85] Wu J. Lin P. C. Guo Y. P. Liu M. D. (2020). The complete chloroplast genome of *Corydalis conspersa*. Mitochondrial DNA B Resour. 5, 1977–1978. 10.1080/23802359.2020.1756944

[B86] Wu Z. Y. Zhuang X. (1982). Study of genus *Corydalis* sect. Oreocapnos M. Popov. Acta Bot. Yunnan. 4, 7–16.

[B87] Wu Z. Y. Zhuang X. Su Z. Y. (1996). The systematic evolution of *Corydalis* in relation to florogenesis and floristic regionalization in the world. Acta Bot. Yunnan. 18, 241–267.

[B88] Wu Z. Y. Zhuang X. Su Z. Y. (1999). *Corydalis* DC in Flora Reipublicae Popularis Sinicae, Tomus 32, ed Delecti Flora Reipublicae Popularis Sinicae Agendae Academiae Sinicae (Beijing: Science Press), 106–479 (in Chinese).

[B89] Xu L. S. Herrando-Moraira S. Susanna A. Galbany-Casals M. Chen Y. S. (2019). Phylogeny, origin and dispersal of *Saussurea* (Asteraceae) based on chloroplast genome data. Mol. Phylogenet. Evol. 141, 106613. 10.1016/j.ympev.2019.106613 31525421

[B90] Xu X. D. Wang D. (2018). *Corydalis ternatifolia* belongs to *C*. sect. *Asterostigmata*, not *C*. sect. *Incisae* (Papaveraceae): evidence from morphological and phylogenetic study. Phytotaxa 382, 193–203. 10.11646/phytotaxa.382.2.4

[B91] Xu X. D. Wang D. (2020). Characterization of the complete chloroplast genome of *Corydalis inopinata* Prain ex Fedde (Papaveraceae). Mitochondrial DNA B Resour. 5, 3302–3303. 10.1080/23802359.2020.1814887 33458142 PMC7782898

[B92] Xu X. D. Wang D. (2021). Comparative chloroplast genomics of *Corydalis* species (Papaveraceae): evolutionary perspectives on their unusual large scale rearrangements. Front. Plant Sci. 11, 600354. 10.3389/fpls.2020.600354 33584746 PMC7873532

[B93] Xu Z. C. Li Z. Ren F. M. Gao R. R. Wang Z. Zhang J. L.. (2022). The genome of *Corydalis* reveals the evolution of benzylisoquinoline alkaloid biosynthesis. Plant J. 1–14. 10.1111/tpj.15788 35476217 PMC7614287

[B94] Yang L. X. Su D. Y. Chang X. Foster C. S. P. Sun L. H. Huang C. H.. (2020). Phylogenomic insights into deep phylogeny of angiosperms based on broad nuclear gene sampling. Plant Commun. 1, 100027. 10.1016/j.xplc.2020.100027 33367231 PMC7747974

[B95] Yu Z. Y. Zhou T. H. Li N. Y. Wang D. (2021). The complete chloroplast genome and phylogenetic analysis of *Corydalis fangshanensis* W.T. Wang ex S.Y. He (Papaveraceae). Mitochondrial DNA B Resour. 6, 3171–3173. 10.1080/23802359.2021.1987172 34746395 PMC8567948

[B96] Zeng C. X. Hollingsworth P. M. Yang J. He Z. S. Zhang Z. R. Li D. Z.. (2018). Genome skimming herbarium specimens for DNA barcoding and phylogenomics. Plant Methods 14, 43. 10.1186/s13007-018-0300-0 29928291 PMC5987614

[B97] Zhai W. Duan X. S. Zhang R. Guo C. C. Li L. Xu G. X.. (2019). Chloroplast genomic data provide new and robust insights into the phylogeny and evolution of the Ranunculaceae. Mol. Phylogenet. Evol. 135, 12–21. 10.1016/j.ympev.2019.02.024 30826488

[B98] Zhang B. Huang R. Z. Hua J. Liang H. Pan Y. M. Dai L. M.. (2016). Antitumor lignanamides from the aerial parts of *Corydalis saxicola*. Phytomedicine 23, 1599–1609. 10.1016/j.phymed.2016.09.006 27823624

[B99] Zhang M. L. Su Z. Y. Lidén M. (2008). *Corydalis* DC., in Flora of China, Volume 7, eds Wu Z. Y. Raven P. H. Hong D. Y. (Beijing: Science Press), 295–427.

[B100] Zhang S. D. Jin J. J. Chen S. Y. Chase M. W. Soltis D. E. Li H. T.. (2017). Diversification of Rosaceae since the Late Cretaceous based on plastid phylogenomics. New Phytol. 214, 1355–1367. 10.1111/nph.14461 28186635

[B101] Zhang Y. Lee J. Liu X. Sun Z. (2019). The first complete chloroplast genome of *Hylomecon japonica* and its phylogenetic position within Papaveraceae. Mitochondrial DNA B Resour. 4, 2349–2350. 10.1080/23802359.2019.1573125 33365538 PMC7687558

[B102] Zhang Y. W. Zhao J. M. Inouye D. W. (2013). Nectar thieves influence reproductive fitness by altering behaviour of nectar robbers and legitimate pollinators in *Corydalis ambigua* (Fumariaceae). J. Ecol. 102, 229–237. 10.1111/1365-2745.12166

[B103] Zhang Z. X. Wang D. Yang X. (2016). The taxonomic position of *Corydalis parviflora* Su and Lidén (Papaveraceae), a genetically distinct species: evidence from cpDNA and nDNA sequences. Biochem. Syst. Ecol. 67, 134–141. 10.1016/j.bse.2016.06.003

[B104] Zhao F. Chen Y. P. Salmaki Y. Drew B. T. Wilson T. C. Scheen A. C.. (2021). An updated tribal classification of Lamiaceae based on plastome phylogenomics. BMC Biol. 19:2. 10.1186/s12915-020-00931-z 33419433 PMC7796571

[B105] Zhu A. D. Guo W. H. Gupta S. Fan W. S. Mower J. P. (2016). Evolutionary dynamics of the plastid inverted repeat: the effects of expansion, contraction, and loss on substitution rates. New Phytol. 209, 1747–1756. 10.1111/nph.13743 26574731

[B106] Zhu Y. Wang D. Zhang L. Y. Liu M. X. (2018). Differential importance of consecutive dispersal phases in two ant-dispersed *Corydalis* species. Nord. J. Bot. 36, e01644. 10.1111/njb.01644

