# Supplementary figures and images for: New Insights Into the Backbone Phylogeny and Character Evolution of Corydalis (Papaveraceae) Based on Plastome Data
Source: Front Plant Sci. 2022 Aug 5;13:926574. doi: 10.3389/fpls.2022.926574 (PMC9389321; doi:10.3389/fpls.2022.926574)

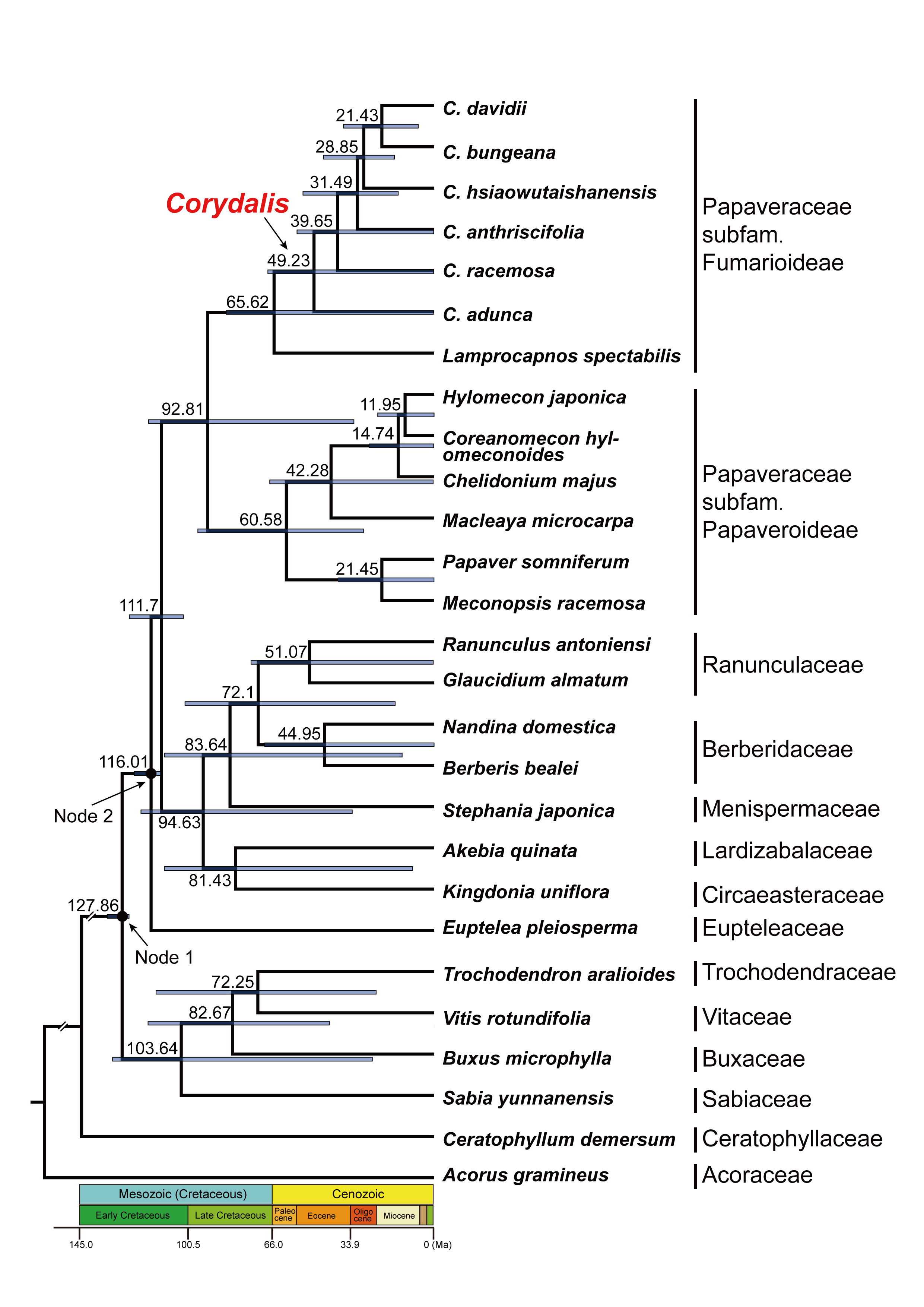

Supplement: Supplementary Figure 1 — The initial chronogram shows the divergence time of Corydalis estimated in BEAST based on plastomes data from six representative Corydalis and 21 outgroup species. Estimated ages are shown near the nodes, and blue bars represent 95% high posterior density. Calibration points (Node 1 and Node 2) were indicated with a black solid circle. [file Image_1.TIF]
