# Supplementary material for: New Insights Into the Backbone Phylogeny and Character Evolution of Corydalis (Papaveraceae) Based on Plastome Data
Source: Front Plant Sci. 2022 Aug 5;13:926574. doi: 10.3389/fpls.2022.926574 (PMC9389321; doi:10.3389/fpls.2022.926574)
Supplement: Supplementary Table 1 — Detailed information for plastome sequencing and assembling results. The column “Assembling result” indicates the numbers of scaffolds that belong to the corresponding part, LSC, IR, and SSC, respectively. For “Total length”, the length of IR was counted two times in the uncircularized plastome. [file Data_Sheet_1.docx]

**Supplementary Table 1** Detailed information for plastome sequencing and assembling results. The column "Assembling result" indicate the numbers of scaffolds that belong to the corresponding part, LSC, IR, and SSC, respectively. For "Total length", the length of IR was counted twice in the un-circularised plastome.

| **Species** | **Raw Base (G)** | **Raw Reads** | **Q30 (%)** | **Clean Base (G)** | **Clean Reads** | **Assembling result**  **(LSC + IR + SSC)** | **Total length (bp)** |
| --- | --- | --- | --- | --- | --- | --- | --- |
| *Corydalis anthriscifolia* Franch. | 5.33 | 17759286 | 90.31 | 5.3 | 17665329 | circular | 184867 |
| *C. balansae* Prain | 5.47 | 18240157 | 90.19 | 5.44 | 18136597 | 4+1+1 | 186683 |
| *C. benecincta* W. W. Sm. | 5.96 | 19871926 | 89.38 | 5.78 | 19270542 | 3+1+1 | 167357 |
| *C. borii* C. E. C. Fisch. | 4.91 | 16374805 | 91.91 | 4.89 | 16303942 | 3+4+1 | 175176 |
| *C. brevirostrata* C. Y. Wu & Z. Y. Su | 5.42 | 18072322 | 89.69 | 5.32 | 17744090 | 1+4+0 | 205407 |
| *C. bungeana* Turcz. | 3.74 | 16940640 | 92.03 | 5.03 | 16775926 | 3+3+1 | 173372 |
| *C. casimiriana* subsp. *brachycarpa* Lidén | 5.41 | 18047429 | 92.29 | 5.38 | 17941118 | 2+3+1 | 170632 |
| *C. caudata* (Lam.) Pers. | 5.69 | 18979521 | 92.47 | 5.65 | 18846072 | 3+2+1 | 184401 |
| *C. cornuta* Royle | 7.05 | 23495495 | 90.11 | 6.94 | 23120559 | 2+4+1 | 182402 |
| *C. crispa* Prain | 5.53 | 18425025 | 90.17 | 5.5 | 18322412 | 3+2+1 | 176714 |
| *C. curviflora* Maxim. | 5.99 | 19969229 | 89.72 | 5.9 | 19682135 | 3+5+1 | 182256 |
| *C. decumbens* (Thunb.) Pers. | 5.72 | 19063902 | 92.16 | 5.66 | 18855864 | 4+2+1 | 176832 |
| *C. edulis* Maxim. | 3.74 | 21356669 | 92.66 | 6.38 | 21267393 | 3+1+1 | 186782 |
| *C. elata* Bur. & Franch. | 5.74 | 19123871 | 89.86 | 5.68 | 18934179 | 3+3+1 | 184558 |
| *C. fargesii* Franch. | 7.21 | 24043332 | 90.03 | 7.13 | 23753715 | 4+2+1 | 173780 |
| *C. hamata* Franch. | 2.96 | 21530868 | 90.05 | 6.41 | 21358617 | 2+2+1 | 188434 |
| *C. hendersonii* Hemsl. | 8.33 | 27770716 | 88.84 | 8.22 | 27402948 | 4+5+1 | 168083 |
| *C. incisa* (Thunb.) Pers. | 5.47 | 23883917 | 92.53 | 7.15 | 23839126 | circular | 213295 |
| *C. jingyuanensis* C. Y. Wu & H. Chuang | 6.24 | 20786367 | 92.18 | 6.2 | 20658710 | 3+3+1 | 181712 |
| *C. livida* Maxim. | 5.07 | 16901227 | 91.94 | 5.05 | 16817587 | 4+2+1 | 189548 |
| *C. longicalcarata* H. Chuang & Z. Y. Su | 5.47 | 12766960 | 93.81 | 3.8 | 12666831 | circular | 195819 |
| *C. melanochlora* Maxim. | 5.68 | 18926410 | 90.2 | 5.6 | 18664694 | 4+3+0 | 187649 |
| *C. minutiflora* C. Y. Wu | 6.27 | 20891394 | 90.03 | 6.22 | 20718811 | 3+3+1 | 182519 |
| *C. mucronata* Franch. | 6.3 | 20990327 | 91.76 | 6.19 | 20645352 | 4+3+1 | 191384 |
| *C. petrophila* Franch. | 5.19 | 17288188 | 90.25 | 5.12 | 17076935 | 2+3+1 | 183257 |
| *C. pseudoadoxa* (C. Y. Wu & H. Chuang) C. Y. Wu & H. Chuang | 2.96 | 22018773 | 91.96 | 6.49 | 21618628 | circular | 190712 |
| *C. pseudoimpatiens* Fedde | 6.53 | 21762084 | 90 | 6.49 | 21627710 | circular | 173581 |
| *C. racemosa* (Thunb.) Pers. | 6.94 | 23134369 | 90.39 | 6.9 | 23005768 | 3+2+1 | 190061 |
| *C. retingensis* Ludlow | 2.96 | 16681549 | 91.7 | 4.98 | 16601150 | 2+3+1 | 205527 |
| *C. stricta* Stephan ex Fisch. | 5.65 | 18838456 | 89.8 | 5.58 | 18589281 | 4+2+1 | 171808 |
| *C. trachycarpa* Maxim. | 6.4 | 21319256 | 91.97 | 6.28 | 20920356 | 2+2+1 | 195326 |
| *C. wuzhengyiana* Z. Y. Su & Lidén | 2.96 | 18747904 | 91.79 | 5.59 | 18649734 | 4+4+1 | 174886 |

**Supplementary Table 2** Optimal partitioning scheme, best fit substitution models, and related parameters for BI analysis. The sign after a gene, "-1", "-2", and "-3" indicate the nucleotide which is corresponding to the first, second or third position of the codon, respectively.

| **Subset** | **Partition names** | **Best Model** | **AICc** | **Sites (no.)** |
| --- | --- | --- | --- | --- |
| 1 | *rps7*-1*, atpB*-1*, atpA*-1 | GTR+I+G | 9183.459 | 1158 |
| 2 | *atpB*-2*, atpA*-2 | GTR+I+G | 6348.6426 | 1003 |
| 3 | *atpB*-3*, petA*-3*, atpA*-3 | GTR+I+G | 31613.041 | 1323 |
| 4 | *atpE*-1*, rps19*-1 | GTR+I+G | 2634.9639 | 225 |
| 5 | *atpE*-2*, petA*-2 | GTR+I+G | 3712.0261 | 453 |
| 6 | *rpl14*-3*, atpE*-3*, ccsA*-3*, petD*-3*, rpl16*-3 | GTR+I+G | 21335.623 | 878 |
| 7 | *atpF*-1*, psbH*-1*, rpoC2*-1*, rps4*-1*, rpl23*-1 | GTR+I+G | 27515.695 | 1910 |
| 8 | *rpl23*-2*, ccsA*-1*, rpl32*-2*, rps19*-2*, atpF*-2*, rps3*-2 | GTR+I+G | 13262.561 | 958 |
| 9 | *psbZ*-3*, psbK*-3*, ycf4*-3*, atpI*-3*, ycf3*-3*, atpF*-3*, rpl33*-3 | GTR+I+G | 20749.562 | 972 |
| 10 | *atpH*-1*, psbI*-1*, psbE*-1*, psbA*-1*, petB*-1 | GTR+I+G | 4018.3982 | 768 |
| 11 | *psbM*-2*, psbF*-2*, atpH*-2 | HKY+I | 432.24243 | 154 |
| 12 | *rps4*-3*, petL*-3*, atpH*-3 | GTR+I+G | 7603.233 | 311 |
| 13 | *psbM*-1*, rps14*-2*, atpI*-1*, rpl2*-2 | GTR+I+G | 5697.771 | 655 |
| 14 | *psbL*-2*, atpI*-2*, psbN*-2 | HKY+I+G | 1700.4202 | 328 |
| 15 | *psaI*-2*, rps16*-2*, ccsA*-2 | GTR+I+G | 5391.5483 | 439 |
| 16 | *cemA*-1*, rpl2*-3 | GTR+G | 8364.341 | 503 |
| 17 | *cemA*-2*, psbL*-3 | GTR+G | 3178.7217 | 267 |
| 18 | *cemA*-3*, psaI*-3 | GTR+G | 6666.038 | 265 |
| 19 | *matK*-1 | GTR+I+G | 11418.887 | 500 |
| 20 | *matK*-2*, rpoA*-2*, rps18*-2 | GTR+I+G | 16385.744 | 938 |
| 21 | *matK*-3 | GTR+G | 14638.816 | 500 |
| 22 | *petA*-1*, rpoB*-1*, rpoC1*-1*, ycf4*-1 | GTR+I+G | 23848.365 | 2251 |
| 23 | *psbA*-2*, psbC*-2*, petB*-2*, psbD*-2 | GTR+I+G | 4870.5225 | 1392 |
| 24 | *petB*-3*, psaA*-3*, psbC*-3 | GTR+G | 29571.227 | 1436 |
| 25 | *psbH*-2*, petD*-1 | K80+I+G | 1834.3617 | 240 |
| 26 | *psbL*-1*, petD*-2 | F81+I | 892.00134 | 205 |
| 27 | *petG*-1*, psbN*-1*, psbC*-1 | GTR+I+G | 3149.185 | 551 |
| 28 | *psbT*-2*, petG*-2*, psbI*-2 | HKY | 446.9045 | 108 |
| 29 | *petG*-3*, rbcL*-3 | GTR+G | 10955.455 | 512 |
| 30 | *rpl14*-1*, rpl36*-1*, petL*-1 | GTR+I+G | 1745.7269 | 190 |
| 31 | *petL*-2*, psbJ*-2*, petN*-2 | HKY+I+G | 621.3169 | 100 |
| 32 | *rps12*-1*, psbE*-2*, petN*-1*, rps12*-2*, psbF*-1 | SYM+I+G | 2198.787 | 397 |
| 33 | *rpl36*-3*, psbM*-3*, petN*-3 | GTR+I | 1839.0573 | 100 |
| 34 | *psbB*-1*, psbJ*-1*, psaB*-1*, psaA*-1 | GTR+I+G | 12858.291 | 2032 |
| 35 | *psbB*-2*, psaB*-2*, psaA*-2 | GTR+I+G | 8273.9375 | 1992 |
| 36 | *psaB*-3*, psbE*-3*, psbD*-3 | GTR+I+G | 22700.855 | 1170 |
| 37 | *psaC*-1 | JC | 339.0017 | 81 |
| 38 | *psaC*-2 | JC | 243.6723 | 81 |
| 39 | *psbN*-3*, psaC*-3*, psbJ*-3 | GTR+G | 2922.6038 | 164 |
| 40 | *ycf2*-3*, psaI*-1*, rpl23*-3 | GTR+G | 30228.99 | 2087 |
| 41 | *psaJ*-1*, rpl16*-2 | SYM+I+G | 1629.3301 | 179 |
| 42 | *psaJ*-2*, psbK*-2*, psbZ*-2 | GTR+I | 1037.9005 | 167 |
| 43 | *psaJ*-3*, psbB*-3 | GTR+G | 12681.649 | 552 |
| 44 | *psbI*-3*, psbA*-3 | GTR+G | 8128.222 | 389 |
| 45 | *psbD*-1 | GTR+I | 1500.751 | 353 |
| 46 | *psbT*-1*, psbF*-3 | GTR+G | 932.7759 | 74 |
| 47 | *rps18*-3*, psbH*-3 | GTR+G | 4221.0664 | 174 |
| 48 | *psbK*-1 | SYM+G | 918.92126 | 61 |
| 49 | *rpl22*-2*, psbT*-3*, rps19*-3 | GTR+G | 5809.2285 | 257 |
| 50 | *rps8*-2*, psbZ*-1*, ycf4*-2 | GTR+G | 3917.5144 | 378 |
| 51 | *rbcL*-1 | GTR+I+G | 3397.1519 | 475 |
| 52 | *rbcL*-2 | GTR+I+G | 2256.144 | 475 |
| 53 | *rps7*-2*, rpl14*-2 | GTR+G | 1839.7816 | 277 |
| 54 | *rpl36*-2*, rpl16*-1 | GTR+I+G | 1919.3999 | 172 |
| 55 | *rpl33*-2*, rps2*-1*, rps8*-1*, rpl2*-1 | GTR+I+G | 7924.3125 | 708 |
| 56 | *rps18*-1*, rpl20*-1 | GTR+G | 4498.298 | 218 |
| 57 | *rpl20*-2 | SYM+I+G | 2052.3247 | 117 |
| 58 | *rpoC2*-3*, rpl20*-3*, rps8*-3 | GTR+I+G | 39466.816 | 1614 |
| 59 | *rps16*-1*, rpl22*-1 | GTR+I+G | 4867.977 | 212 |
| 60 | *rpl22*-3*, rps16*-3*, rpl32*-3 | GTR+G | 9408.675 | 266 |
| 61 | *rpl32*-1 | GTR+G | 1022.44836 | 54 |
| 62 | *rps7*-3*, rps12*-3*, rpl33*-1 | GTR+G | 3442.7249 | 344 |
| 63 | *rpoA*-1*, rps11*-1 | GTR+G | 8643.902 | 475 |
| 64 | *rps11*-3*, rps3*-3*, rpoA*-3 | GTR+I+G | 19065.662 | 693 |
| 65 | *ycf3*-2*, rpoC1*-2*, rps4*-2*, rps2*-2*, rpoB*-2 | GTR+I+G | 22613.234 | 2350 |
| 66 | *rpoC1*-3*, rps2*-3*, rpoB*-3 | GTR+G | 41455.977 | 1983 |
| 67 | *rpoC2*-2 | GTR+I+G | 18621.006 | 1365 |
| 68 | *rps11*-2 | GTR+G | 1813.9058 | 138 |
| 69 | *ycf2*-1*, rps14*-1 | GTR+G | 27246.379 | 2062 |
| 70 | *rps14*-3*, rps3*-1 | GTR+G | 6068.777 | 318 |
| 71 | *ycf1*-1 | GTR+I+G | 47978.03 | 1714 |
| 72 | *ycf1*-2 | GTR+I+G | 42797.86 | 1714 |
| 73 | *ycf1*-3 | GTR+I+G | 54535.164 | 1714 |
| 74 | *ycf2*-2 | GTR+I+G | 25721.8 | 1962 |
| 75 | *ycf3*-1 | F81+G | 1545.7301 | 168 |

**Supplementary Table 3** Optimal partitioning scheme and related parameters using GTR+I+G substitution model for ML analysis. The sign after a gene, "-1", "-2", or "-3" indicate the nucleotide which is corresponding to the first, second or third position of the codon, respectively.

| **Subset** | **Partition names** | **Best Model** | **AICc** | **Sites (no.)** |
| --- | --- | --- | --- | --- |
| 1 | *rps7*-1*, rpl36*-2*, atpA*-1*, atpB*-1 | GTR+I+G | 9492.77 | 1195 |
| 2 | *atpA*-2*, atpB*-2 | GTR+I+G | 6348.6426 | 1003 |
| 3 | *psbB*-3*, petA*-3*, atpA*-3*, atpB*-3*, petB*-3*, psaJ*-3 | GTR+I+G | 49134.21 | 2090 |
| 4 | *atpE*-1*, rps19*-1*, rpl33*-1 | GTR+I+G | 3383.5037 | 291 |
| 5 | *atpE*-2*, petA*-2 | GTR+I+G | 3712.0261 | 453 |
| 6 | *petD*-3*, rpl16*-3*, petL*-3*, atpH*-3*, rpl14*-3*, atpE*-3*, ccsA*-3 | GTR+I+G | 23996.639 | 990 |
| 7 | *atpF*-1*, rps14*-1*, rpl23*-2 | GTR+I+G | 4711.0605 | 373 |
| 8 | *ccsA*-1*, rpoA*-2*, atpF*-2*, rps3*-2 | GTR+I+G | 15273.641 | 1060 |
| 9 | *rpl33*-3*, atpF*-3*, ycf3*-3*, psbZ*-3*, psbK*-3*, atpI*-3*, ycf4*-3 | GTR+I+G | 20749.562 | 972 |
| 10 | *atpH*-1*, psbA*-1*, psbC*-1*, petG*-1*, psbN*-1*, psbI*-1*, petB*-1*, psbE*-1 | GTR+I+G | 7169.885 | 1319 |
| 11 | *psbF*-2*, atpH*-2*, psbD*-2*, petB*-2*, psbA*-2*, psaC*-2*, psbC*-2 | GTR+I+G | 5472.2866 | 1593 |
| 12 | *psbM*-1*, rps14*-2*, rpl2*-2*, atpI*-1*, petD*-1 | GTR+I+G | 6800.152 | 822 |
| 13 | *atpI*-2*, psbL*-2*, petG*-2*, psbI*-2*, psbM*-2*, psbT*-2 | GTR+I+G | 2009.7028 | 427 |
| 14 | *rpl20*-2*, psbH*-2*, ccsA*-2 | GTR+I+G | 6490.9316 | 511 |
| 15 | *rpl2*-3*, rps18*-2*, cemA*-1 | GTR+I+G | 10618.53 | 604 |
| 16 | *psbL*-3*, cemA*-2 | GTR+I+G | 3180.881 | 267 |
| 17 | *psaI*-3*, cemA*-3 | GTR+I+G | 6668.198 | 265 |
| 18 | *matK*-1 | GTR+I+G | 11418.887 | 500 |
| 19 | *psbF*-3*, matK*-2 | GTR+I+G | 9566.501 | 539 |
| 20 | *matK*-3 | GTR+I+G | 14640.896 | 500 |
| 21 | *rpoB*-1*, rpoC1*-1*, rpl33*-2*, petA*-1 | GTR+I+G | 22182.742 | 2133 |
| 22 | *psbB*-2*, petD*-2*, psaA*-2*, psaB*-2 | GTR+I+G | 8976.752 | 2159 |
| 23 | *rbcL*-3*, petG*-3 | GTR+I+G | 10956.5 | 512 |
| 24 | *rpl14*-1*, petL*-1*, rpl36*-1 | GTR+I+G | 1745.7269 | 190 |
| 25 | *psbN*-2*, psbJ*-2*, petN*-2*, petL*-2 | GTR+I+G | 858.0956 | 143 |
| 26 | *rps12*-1*, psbT*-1*, psbF*-1*, petN*-1*, rps12*-2 | GTR+I+G | 2157.2627 | 349 |
| 27 | *psbE*-3*, psbD*-3*, rpl36*-3*, petN*-3*, psbM*-3 | GTR+I+G | 9555.19 | 536 |
| 28 | *psaA*-1*, psbB*-1*, psbJ*-1 | GTR+I+G | 8668.938 | 1298 |
| 29 | *psaB*-3*, psaA*-3*, psbC*-3 | GTR+I+G | 39719.305 | 1955 |
| 30 | *psbL*-1*, psaC*-1*, psbE*-2*, psaB*-1 | GTR+I+G | 5121.1343 | 936 |
| 31 | *psbJ*-3*, psbN*-3*, psaC*-3 | GTR+I+G | 2924.8743 | 164 |
| 32 | *ycf2*-3*, rpl23*-3*, psaI*-1 | GTR+I+G | 30231.012 | 2087 |
| 33 | *psaI*-2*, psbK*-1*, rps16*-2 | GTR+I+G | 2616.5637 | 179 |
| 34 | *psaJ*-1*, rpl16*-2 | GTR+I+G | 1635.3707 | 179 |
| 35 | *psbK*-2*, psbZ*-2*, psaJ*-2 | GTR+I+G | 1040.4861 | 167 |
| 36 | *psbA*-3*, psbI*-3 | GTR+I+G | 8128.8755 | 389 |
| 37 | *psbD*-1 | GTR+I+G | 1502.7196 | 353 |
| 38 | *ycf4*-2*, psbZ*-1*, rps8*-2*, psbH*-1*, ycf4*-1 | GTR+I+G | 7154.018 | 635 |
| 39 | *rps18*-3*, psbH*-3 | GTR+I+G | 4223.322 | 174 |
| 40 | *rpl22*-2*, psbT*-3*, rps19*-3 | GTR+I+G | 5810.678 | 257 |
| 41 | *rbcL*-1 | GTR+I+G | 3397.1519 | 475 |
| 42 | *rbcL*-2 | GTR+I+G | 2256.144 | 475 |
| 43 | *rpl14*-2*, rps7*-2 | GTR+I+G | 1841.5144 | 277 |
| 44 | *rps2*-1*, rpl16*-1 | GTR+I+G | 4476.6333 | 371 |
| 45 | *rpl2*-1*, rps8*-1 | GTR+I+G | 4336.8867 | 406 |
| 46 | *rpl20*-1*, rpl32*-1 | GTR+I+G | 3095.0762 | 171 |
| 47 | *rpoC2*-3*, rps4*-3*, rpl20*-3*, rps8*-3 | GTR+I+G | 44411.96 | 1813 |
| 48 | *rps18*-1*, rps16*-1*, rpl22*-1 | GTR+I+G | 7294.1035 | 313 |
| 49 | *rpl22*-3*, rps16*-3*, rpl32*-3 | GTR+I+G | 9409.776 | 266 |
| 50 | *rpoC2*-1*, rpl23*-1*, rps4*-1 | GTR+I+G | 24284.447 | 1653 |
| 51 | *rpl32*-2*, rps19*-2 | GTR+I+G | 2017.7104 | 146 |
| 52 | *rps11*-1*, rpoA*-1 | GTR+I+G | 8645.8125 | 475 |
| 53 | *rpoA*-3*, rps3*-3*, rps11*-3 | GTR+I+G | 19065.662 | 693 |
| 54 | *ycf3*-2*, rpoC1*-2*, rps4*-2*, rpoB*-2*, rps2*-2 | GTR+I+G | 22613.234 | 2350 |
| 55 | *rpoC1*-3*, rpoB*-3*, rps2*-3 | GTR+I+G | 41457.32 | 1983 |
| 56 | *rpoC2*-2 | GTR+I+G | 18621.006 | 1365 |
| 57 | *rps11*-2 | GTR+I+G | 1814.8435 | 138 |
| 58 | *rps7*-3*, rps12*-3 | GTR+I+G | 2696.0962 | 278 |
| 59 | *rps3*-1*, rps14*-3 | GTR+I+G | 6069.918 | 318 |
| 60 | *ycf1*-1 | GTR+I+G | 47978.03 | 1714 |
| 61 | *ycf1*-2 | GTR+I+G | 42797.86 | 1714 |
| 62 | *ycf1*-3 | GTR+I+G | 54535.164 | 1714 |
| 63 | *ycf2*-1 | GTR+I+G | 26037.504 | 1962 |
| 64 | *ycf2*-2 | GTR+I+G | 25721.8 | 1962 |
| 65 | *ycf3*-1 | GTR+I+G | 1546.8915 | 168 |

**Supplementary Table 4** Ages of *Corydalis* clades estimated using BEAST. Age represents stem age (S) or the age of split from most recent common ancestors, unless otherwise indicated (crown age, C).

| **Taxon/Clade** | **Mean age** | **95% HPD min** | **95% HPD max** |
| --- | --- | --- | --- |
| Ranunculales (C) | 116.01 | 112.04 | 122.84 |
| Papaveraceae | 111.70 | 102.75 | 124.92 |
| Papaveraceae (C) | 92.81 | 32.90 | 117.06 |
| subfam. Fumarioideae (C) | 65.65 | 63.72 | 67.60 |
| *Corydalis* (C) | 49.08 | 47.18 | 51.03 |
| Clade Ⅰ | 49.08 | 47.18 | 51.03 |
| Clade Ⅱ | 39.99 | 33.54 | 45.96 |
| Clade Ⅲ | 33.16 | 26.77 | 39.71 |
| Clade Ⅳ | 31.43 | 25.19 | 37.78 |
| Clade Ⅴ | 28.36 | 22.46 | 34.25 |
| Clade Ⅵ | 28.36 | 22.46 | 34.25 |
| **Clade Ⅰ** |  |  |  |
| *C. stricta* (sect. *Strictae*) | 6.26 | 2.10 | 11.60 |
| *C. adunca* (sect. *Strictae*) | 6.26 | 2.10 | 11.60 |
| **Clade Ⅱ** |  |  |  |
| *C. racemosa* (sect. *Cheilanthifoliae*) | 22.29 | 12.44 | 32.93 |
| *C. balansae* (sect. *Sophorocapnos*) | 7.93 | 3.59 | 13.14 |
| *C. saxicola* (sect. *Thalictrifoliae*) | 2.63 | 0.93 | 4.68 |
| *C. edulis* (sect. *Aulacostigma*) | 2.63 | 0.93 | 4.68 |
| **Clade Ⅲ** |  |  |  |
| *C. longicalcarata* (sect. *Archaeocapnos*) | 26.61 | 17.86 | 34.97 |
| *C. anthriscifolia* (sect. *Archaeocapnos*) | 26.61 | 17.86 | 34.97 |
| **Clade Ⅳ** |  |  |  |
| Split Ⅳ-1 | 25.49 | 18.34 | 32.47 |
| *C. hsiaowutaishanensis* (sect. *Dactylotuber*) | 22.09 | 14.33 | 29.67 |
| *C. retingensis* (sect. *Oocapnos*) | 22.09 | 14.33 | 29.67 |
| Split Ⅳ-2 | 23.62 | 16.60 | 30.53 |
| *C. caudata* (sect. *Corydalis*) | 13.09 | 6.03 | 20.42 |
| *C. benecincta* (sect. *Benecinctae*) | 13.09 | 6.03 | 20.42 |
| *C. livida* (sect. *Flaccidae*) | 21.72 | 14.72 | 28.63 |
| *C. decumbens* (sect. *Duplotuber*) | 21.72 | 14.72 | 28.63 |
| **Clade Ⅴ** |  |  |  |
| *C. davidii* (sect. *Davidianae*) | 22.32 | 16.99 | 27.61 |
| Split Ⅴ-1 | 19.24 | 14.48 | 24.29 |
| *C. pseudoimpatiens* (*sibirica group*) | 9.25 | 4.47 | 14.61 |
| *C. fargesii* (*ochotensis group*) | 9.25 | 4.47 | 14.61 |
| Split Ⅴ-2 | 17.97 | 13.37 | 22.83 |
| *C. inopinata* (sect. *Mucroniferae*) | 12.45 | 6.96 | 17.89 |
| *C. trachycarpa* (sect. *Trachycarpae*) | 12.45 | 6.96 | 17.89 |
| *C. crispa* (sect. *Radicosae*) | 15.67 | 11.30 | 20.25 |
| Split Ⅴ-3 | 12.75 | 8.76 | 16.92 |
| *C. casimiriana* subsp. *brachycarpa* (sect. *Himalayanae*) | 11.21 | 7.33 | 15.21 |
| *C. borii* (sect. *Geraniifoliae*) | 8.13 | 4.47 | 12.01 |
| *C. cornuta* (sect. *Ramososibiricae*) | 8.13 | 4.47 | 12.01 |
| *C. hendersonii* (sect. *Latiflorae*) | 7.06 | 3.74 | 10.70 |
| *C. dasyptera* (sect. *Chrysocapnos*) | 3.03 | 1.10 | 5.33 |
| *C. wuzhengyiana* (sect. *Chrysocapnos*) | 3.03 | 1.10 | 5.33 |
| **Clade Ⅵ** |  |  |  |
| *C. bungeana* (sect. *Chinenses*) | 22.43 | 16.52 | 28.34 |
| Split Ⅵ-1 | 18.97 | 13.55 | 24.48 |
| *C. brevirostrata* (sect. *Vermiculares*) | 17.02 | 11.61 | 22.41 |
| *C. temulifolia* (sect. *Asterostigma*) | 13.60 | 8.56 | 18.68 |
| *C. incisa* (sect. *Incisae*) | 9.88 | 5.27 | 14.73 |
| *C. mucronata* (sect. *Mucronatae*) | 9.88 | 5.27 | 14.73 |
| *C. petrophila* (sect. *Priapos*) | 9.66 | 5.96 | 13.70 |
| Split Ⅵ-2 | 6.65 | 4.16 | 9.37 |
| *C. elata* (sect. *Elatae*) | 3.41 | 1.39 | 5.69 |
| *C. jingyuanensis* (sect. *Ellipticarpae*) | 3.41 | 1.39 | 5.69 |
| *C. hamata* (sect. *Hamatae*) | 6.46 | 3.99 | 9.15 |
| Split Ⅵ-3 | 4.42 | 2.60 | 6.47 |
| *C. curviflora* (ser. *Curviflorae*) | 2.86 | 1.31 | 4.52 |
| *C. minutiflora* (ser. *Kokianae*) | 2.86 | 1.31 | 4.52 |
| *C. pseudoadoxa* (ser. *Fusiformes*) | 3.40 | 1.69 | 5.33 |
| *C. melanochlora* (ser. *Clavatae*) | 3.40 | 1.69 | 5.33 |
